# Mechanisms of temozolomide resistance in glioblastoma - a comprehensive review

**DOI:** 10.20517/cdr.2020.79

**Published:** 2021-03-19

**Authors:** Neha Singh, Alexandra Miner, Lauren Hennis, Sandeep Mittal

**Affiliations:** ^1^Division of Neurosurgery, Virginia Tech Carilion School of Medicine, Roanoke, VA 24014, USA.; ^2^Fralin Biomedical Research Institute at VTC, Roanoke, VA 24014, USA.; ^3^Carilion Clinic - Neurosurgery, Roanoke, VA 24014, USA.

**Keywords:** Glioblastoma, temozolomide, chemoresistance, molecular mechanisms

## Abstract

Glioblastoma (GBM) is the most common primary malignant brain tumor in adults and has an exceedingly low median overall survival of only 15 months. Current standard-of-care for GBM consists of gross total surgical resection followed by radiation with concurrent and adjuvant chemotherapy. Temozolomide (TMZ) is the first-choice chemotherapeutic agent in GBM; however, the development of resistance to TMZ often becomes the limiting factor in effective treatment. While O6-methylguanine-DNA methyltransferase repair activity and uniquely resistant populations of glioma stem cells are the most well-known contributors to TMZ resistance, many other molecular mechanisms have come to light in recent years. Key emerging mechanisms include the involvement of other DNA repair systems, aberrant signaling pathways, autophagy, epigenetic modifications, microRNAs, and extracellular vesicle production. This review aims to provide a comprehensive overview of the clinically relevant molecular mechanisms and their extensive interconnections to better inform efforts to combat TMZ resistance.

## Introduction

### The clinical landscape of glioblastoma

Glioblastoma (GBM) is the most common malignant brain tumor in adults, accounting for approximately 15% of all central nervous system tumors and about 45% of primary malignant brain tumors^[1]^. Despite extensive research efforts to better understand and treat these tumors, the prognosis for GBM remains extremely poor with a median overall survival of approximately 15 months and progression-free survival of only 6 months^[2,3]^. Current standard-of-care consists of maximal safe surgical resection followed by radiation with concomitant and adjuvant chemotherapy^[4]^. Temozolomide (TMZ) has been widely used as the standard chemotherapy for newly-diagnosed GBM since its initial FDA approval in 2005 and the subsequent widespread use of the Stupp regimen^[5]^. In their landmark paper, Stupp *et al*.^[5]^ showed that the addition of TMZ chemotherapy to radiation led to a 2-month increase in median overall survival, and this remains one of the most significant enhancements in GBM survival achieved by a novel chemotherapeutic drug to date. The anti-angiogenic agent bevacizumab (Avastin®) has also become a popular second-line therapy in the past decade. While studies have shown that it does not improve overall survival in comparison to standard TMZ plus radiotherapy, it has demonstrated utility in quality of life improvement and symptom management^[6,7]^. Interestingly, a recent study suggests that bevacizumab may affect chemotherapeutic delivery through alteration of perfusion dynamics, suggesting that bevacizumab may play a role in TMZ delivery and preventing resistance^[8]^.

In addition to traditional chemotherapy and radiation, there are also a number of innovative treatments that have come into recent use for GBM, including carmustine wafers (Gliadel®)^[9,10]^, an intracranially implanted local chemotherapy, as well as low-intensity alternating electrical tumor treating fields (Optune® therapy) with anti-proliferative properties^[11-14]^. These treatment options have been shown to confer significant benefit to some patients and have the advantage of being well-tolerated with few side effects as they are localized (Gliadel®) or non-invasive (Optune®) therapies^[9-14]^. Tumor treating fields (TTFields) in particular show true potential for incorporation into standard-of-care based on a recent clinical trial of TTFields plus TMZ, which produced a median overall survival of 20.9 months compared to TMZ alone^[14,15]^. However, even with the addition of new therapies, TMZ remains the mainstay of high-grade glioma treatment, so a thorough understanding of its functionality and prevention of resistance development are of great importance to successful GBM treatment.

Following the 2016 update to the World Health Organization Classification of Tumors of the Central Nervous System^[16]^, there has been an increasing effort to integrate molecular tumor characteristics into GBM diagnosis and treatment in addition to histological analysis. Isocitrate dehydrogenase (IDH) and O6-methylguanine-DNA methyltransferase (MGMT) status are of particular importance in GBM, with mutant IDH and methylated MGMT being associated with better prognosis^[17]^. However, only a small portion of tumors are IDH-mutant (~10%) or have methylated MGMT (~50%), and MGMT status in particular may change over the course of treatment^[18-20]^. More nuanced molecular classification of tumors has also created the opportunity to understand how different tumor subtypes respond to TMZ treatment, as demonstrated by the finding that although IDH wild-type tumors have a worse overall prognosis, they may have an improved response to TMZ^[17,20,21]^. This demonstrates the importance of a more refined understanding of TMZ resistance mechanisms in the advancement of GBM stratification and treatment.

### History of temozolomide in glioblastoma treatment

TMZ is part of the larger class of DNA alkylating agents that were first discovered to have anti-tumorigenic effects in the 1940s and quickly became the first drugs to be used as chemotherapeutics. However, TMZ specifically was not synthesized until the late 1970s and was approved for medical use in Europe and the United States in the early 2000s^[22]^. TMZ (brand names: Temodar, Temodal) is an imidazotetrazine lipophilic prodrug that is able to cross the blood-brain barrier and can therefore be administered orally. It is stable at acidic pH and is activated at physiological pH through conversion to the metabolite 5-(3-methyltriazen-1-yl) imidazole-4-carboxamide (MTIC)^[5]^. MTIC is then hydrolyzed to produce methyldiazonium ions which are electrophilic methylated molecules that cause DNA damage. Negatively charged DNA acts as a nucleophile and the methyl group from the methyldiazonium ion is transferred to DNA, resulting in multiple DNA adducts that create opportunity for mismatched base pairing and ultimately cytotoxicity^[22,23]^.

As a monofunctional alkylating agent, TMZ affects single strands of DNA at specific sites; it preferentially methylates DNA at the N7 position of guanine, O3 position of adenine and O6 position of guanine^[24]^. Alkylation of the O6 site on guanine leads to the formation of O6-methylguannine adducts and results in the insertion of thymine residues instead of cytosine. These unrepairable mutations induce the formation of single- and double-stranded DNA breaks resulting in cell cycle arrest at G2/M and apoptosis^[25]^.

### Development of temozolomide resistance

TMZ has become a cornerstone of GBM treatment, but it is unfortunately also a key factor in tumor resistance and recurrence. Due to the widespread exposure to TMZ and highly heterogeneous and mutation prone nature of GBM, it is quite common for these lethal tumors to develop resistance to TMZ. Unfortunately, over 50% of GBM patients treated with TMZ do not respond to the therapy, yet there are limited predictive markers for TMZ response beyond MGMT status^[22,26]^. Understanding and combating TMZ resistance is further complicated by the fact that resistance can be either inherently characteristic of certain tumors or acquired after initial treatment^[27]^.

TMZ resistance is a clinically meaningful and substantial obstacle that must be overcome for successful treatment of GBM^[28]^. Thus, an in-depth understanding of the cellular populations that drive resistance and the molecular mechanisms that underlie its development and potentiation is of paramount importance. In this review, we present an organized and comprehensive overview of the pathways and mechanisms involved in the complex resistance of GBM tumors to TMZ to better inform future research and treatment design.

## The role of glioma stem cells in resistance

**Figure 1:**
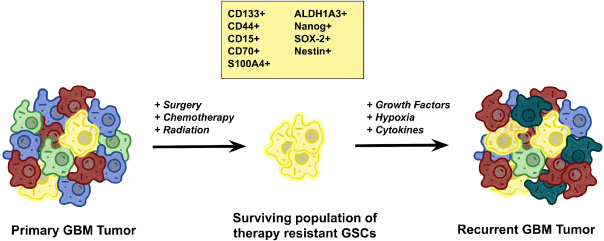
The role of glioma stem cells (GSCs) in tumorigenesis. The heterogeneous nature of glioblastoma (GBM) tumors creates an opportunity for the evolutionary selection of particular clonal cell populations with growth advantages and propagates advantageous mutations. In particular, a small population of quiescent cells with stem-like properties, GSCs, has been shown to be particularly resistant to temozolomide treatment in GBM and these cells are thought to act as the recurrent tumor-initiating cells, which are responsible for regrowth of tumors after initial treatment. While the criteria for defining GSCs are still evolving, the most commonly used markers for GSCs include CD133+, CD44+, CD15+, CD70+, S100A4, ALDH1A3, Nanog, SOX-2, and Nestin. These stem cell characteristics are enhanced by many of the molecular mechanisms of temozolomide resistance in GBM as well as being intrinsic drivers of resistance. Modified from Xie *et al*.^[28]^

The troubling phenomenon of TMZ resistance is thought to be driven primarily by a unique population of undifferentiated and highly tumorigenic cancer stem cells known as glioma stem cells (GSCs; Figure 1). Investigation into this population of therapy-resistant cells was first prompted by the observation of a subset of cells in glial tumors capable of forming heterogeneous clonal populations^[29,30]^. It was also demonstrated that radiation and chemotherapy regimens produced enriched populations of stem-like CD133^+^ cells, thought to be mediated by an upregulation in DNA repair mechanisms^[31]^. To better identify GSCs, there has been a significant effort to establish reliable GSC markers. Although still somewhat debated, the most widely accepted and utilized markers of GSCs include CD133, CD44, CD15, CD70, S100A4, ALDH1A3, Nanog, SOX-2, and Nestin^[32]^.

With advancing technology, there have been many elegant studies that further interrogate the renewal capacity and evolutionary patterns of GSCs. Whole genome and exome sequencing of paired primary and recurrent tumors reveals shared mutations between the genomic profiles that can be traced to a population of resistant GSCs^[33,34]^; and fate mapping studies using genetic barcoding have shown that chemotherapy produces an evolutionary selective pressure resulting in the expansion of drug-resistant GSCs^[35,36]^. Taken together, these results suggest that GSCs are at the core of TMZ resistance and must be specifically targeted to prevent recurrence.

While GSCs comprise as few as 1% of cells in any given GBM tumor, they are responsible for a majority of tumor recurrences due to their ability to regenerate tumor heterogeneity which allows for the development of resistance to both standard chemotherapeutic agents as well as targeted therapies^[37]^. For this reason, many of the molecular mechanisms uncovered in the recent studies discussed here have been determined in the context of GSC populations. This has become standard practice in most modern GBM research and is prudent in overcoming the crucial contribution of GSCs to TMZ resistance.

## DNA repair and temozolomide resistance

### MGMT

**Figure 2:**
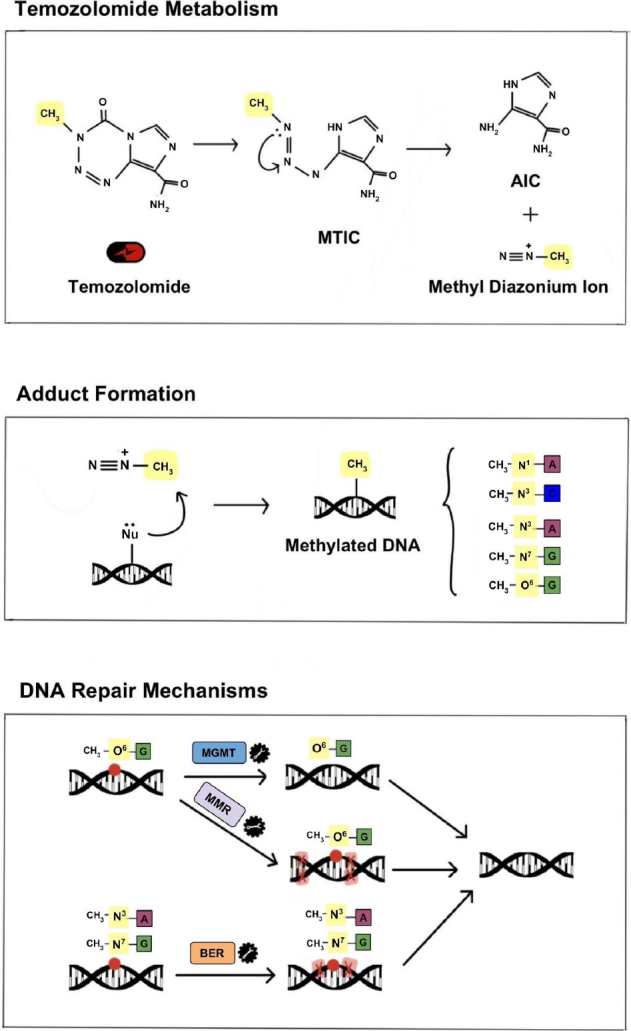
Mechanism of TMZ metabolism, DNA damage and DNA repair. Under physiological conditions TMZ is metabolized to MTIC and then to its active form, a methyl diazonium ion. The electrophilic methyl diazonium ion acts as a methyl donor, transferring its methyl group to negatively charged DNA and creating DNA adducts. This alkylating activity occurs preferentially at N7 of guanine, O3 of adenine and O6 of guanine and, if left unrepaired, results in improper base pairing and single and double-stranded DNA breaks. The primary endogenous DNA repair mechanisms that counteract the DNA damage caused by TMZ, and are thus commonly implicated in TMZ resistance, include MGMT, BER and MMR. TMZ: temozolomide; MTIC: metabolite 5-(3-methyltriazen-1-yl) imidazole-4-carboxamide; MGMT: O6-methylguanine-DNA methyltransferase; BER: base excision repair; MMR: mismatch repair

MGMT is often considered the most important contributor to TMZ resistance due to its direct role in counteracting DNA alkylation damage [Figure 2]. MGMT is an endogenous DNA repair enzyme that helps maintain genomic stability through mismatch repair. Under conditions of TMZ treatment, MGMT can remove the methyl group in O6-methylguanine thereby neutralizing the drug-induced DNA damage and reducing the overall efficacy of TMZ. Therefore, MGMT expression, which is determined by CpG methylation status of the *MGMT* gene promoter region, is an important factor in TMZ treatment response. Hypermethylation of the *MGMT* promoter results in decreased expression of the MGMT protein and has been shown to correlate with prolonged survival in GBM patients. In contrast, unmethylated tumors (with increased MGMT activity) commonly exhibit resistance to TMZ. Therefore, the epigenetic status of *MGMT* has been established as a surrogate marker of intrinsic resistance to TMZ^[38,39]^.

Additionally, there is mounting evidence from meta-analysis studies suggesting that MGMT status may be susceptible to change throughout a tumor’s treatment, progression or recurrence^[38]^. It has been observed that tumors with initial MGMT methylation have a decreased methylation ratio upon recurrence after treatment with TMZ, suggesting that the reduction in MGMT promoter methylation is a mechanism for acquiring therapeutic resistance to TMZ^[40]^. A deeper understanding of the mechanisms that prompt change in MGMT methylation and their contribution to TMZ resistance will be fundamental to comprehensive GBM treatment. Recent emerging evidence from clinical trials suggests that a combination treatment of lomustine and TMZ may improve overall survival when used as a first-line treatment for patients with MGMT methylation, further demonstrating the clinical importance of targeting and characterizing DNA repair enzymes early in GBM treatment^[41]^.

### Base excision repair

Base excision repair (BER) is responsible for repairing single nucleotide modifications, and its mechanism involves several enzymatic reactions carried out by glycosylase, endonuclease, polymerase and DNA ligase^[22,24]^. The vast majority (> 90%) of N^7^-guanine and N^3^-adenine methylation that is induced by TMZ is recognized and rapidly repaired by BER mechanisms^[42,43]^. Several proteins involved in the BER pathway have been associated with promoting resistance to TMZ and confer poor prognosis in patients; these include DNA glycosylase MPG, stem cell factor high-mobility group A2, as well as, DNA polymerase-β (pol-B) which protects from DNA-induced cytotoxicity via its lyase activity^[42,44,45]^. Another protein involved in the BER pathway is poly (ADP-ribose) polymerase 1 or PARP1. This protein recognizes breaks in single-stranded DNA and protects cells from accumulating too many apurinic/apyrimidinic sites^[46,47]^. Tang *et al*.^[42]^ found that the elimination of pol-B combined with administration of PARP1 inhibitors increases TMZ sensitivity resulting from the build-up of damaged DNA. Interestingly, PARP1 also exhibits an alternate role; excessive activation of PARP1 has been shown to promote DNA damage in addition to depletion of nicotinamide adenine dinucleotide (NAD+) and adenosine triphosphate (ATP)^[48-52]^. These findings suggest that mutations in components that either regulate or participate in the BER pathway increase the potential of TMZ cytotoxicity.

### Mismatch repair

Mismatch repair (MMR) is a DNA repair mechanism that works by correcting any mismatched nucleotide base pairings; this process takes place during DNA synthesis. As mentioned earlier, TMZ functions to produce O^6^-MeG which is incorrectly paired with thymine during DNA synthesis^[53]^. Under normal conditions, MMR is activated and generates breaks in the newly synthesized DNA strand. Repeated cycles of DNA breaks generated by the MMR system in response to TMZ-induced DNA damage results in irreparable DNA strands and eventually apoptosis^[54]^. This mechanism reveals the need for intact MMR mechanisms for TMZ-induced cytotoxicity.

MMR has been implicated in TMZ resistance through the development of MMR mutations both *de novo* and in response to standard chemotherapeutic treatment; these MMR mutations have been found to lead to hypermutation in recurrent tumors, particularly in the setting of initial MGMT methylation^[55]^. Interestingly, MMR proteins also tend to display inverse expression in relation to MGMT, with the combination of methylated MGMT and high MMR activity conferring the best response to TMZ^[56]^. This relationship may be exploited in the development of TMZ resistance, where a protective DNA repair genotype may be counteracted by a mutation in a different repair system. More detailed MMR characterization is being explored in GBM with the hope of identifying specific MMR proteins as additional prognostic markers^[57,58]^. There have also been recent efforts to develop a more comprehensive assessment of DNA repair capacity that accounts for MGMT, MMR, BER, nucleotide excision repair and homologous recombination (HR)^[59]^. Although not yet widely implemented, these efforts may help to more accurately predict chemoresistance.

### Interplay of DNA repair and molecular pathway mutations

Many specific mutations or pathway perturbances have been found to alter the efficacy of these DNA integrity control mechanisms. For example, epidermal growth factor receptor variant III (EGFRvIII), a prominent GBM mutation, has been shown to sensitize cells to TMZ through upregulation of MMR in MGMT methylated tumors^[60]^. In MGMT unmethylated tumors which are able to repair and evade TMZ-induced damage, modulation of other tumorigenic pathways has shown benefit in combating resistance and may suggest mechanistic relationships with MGMT.

Regulatory relationships have also been found between well-known oncogenic pathways and DNA repair machinery, creating opportunities for the development of TMZ resistance via mutations in these pathways. In particular, there is evidence of a direct connection between DNA repair and major pathways such as PI3K/Akt and STAT3. For example, it has been shown that inhibition of nuclear factor kappa-B subunit epsilon (IKBKE), an activator of the Akt/nuclear factor kappa-B (NF-κB) pathway, increases TMZ resistance by upregulating MGMT activity^[61]^; and MGMT status has been found to correlate with PI3K/Akt activity^[62]^.

The interaction between MGMT function and other proteins and pathways highlights the interplay between many of the mechanisms discussed below and demonstrates the important concept that compensatory pathway upregulation is a common mechanism for resistance that must be taken into account when designing novel treatments^[38]^.

## Key molecular pathways in temozolomide resistance

### Akt

In addition to DNA repair enzymes contributing to TMZ resistance, the dysregulation of specific molecular pathways is another cornerstone of TMZ resistance. In particular, the PI3K/Akt pathway has been extensively studied in GBM, because of the common mutation and amplification of the pathway’s initiating receptor tyrosine kinases (RTKs)^[63]^. One of the most well-known RTK aberrations is the amplification of EGFR, present in ~60% of GBMs, which results in dysregulation of downstream PI3K/Akt pathways^[64-66]^. Furthermore, ~50% of GBMs with EGFR overexpression harbor a unique mutant variant, EGFRvIII, which is a constitutively active version of the receptor that exhibits ligand-independent activation^[67]^. EGFR amplification and EGFRvIII have been implicated in many resistance mechanisms and demonstrate the significance of perturbing RTK function in GBM.

Upon activation by ligands, RTKs signal through two major downstream pathways, Akt and MAPK. Dysregulation of the PI3K/Akt pathway occurs in up to 88% of GBM tumors, and mutations in the MAPK/Erk pathway are found in the vast majority (> 90%) of tumors^[64]^. Akt, also known as protein kinase B, is a serine/threonine kinase that plays a crucial role in promoting chemoresistance in GBM cells^[68]^. Several downstream targets of Akt have been found to be implicated in specific mechanisms of TMZ resistance, including pyruvate dehydrogenase kinase 1 (PDK1), hypoxia inducible factor 1 (HIF-1), forkhead O3 (FoxO3a), NF-kB, and other apoptotic regulators.

GBM, like many other solid tumors, preferentially utilizes glycolytic pathways for metabolism over oxidative phosphorylation, a shift referred to as the Warburg effect. PDK1 is thought of as the gatekeeper of glucose oxidation and functions to inactivate pyruvate dehydrogenase (PDH) by phosphorylation on the E1-alpha subunit, inactivating the complex and preventing the production of acetyl-CoA, thereby inhibiting the metabolism of pyruvate in the Krebs cycle^[69]^. The inactivation of PDH is supported by increased expression of PDK1 in GBM cells^[70-72]^.

Recent studies have shown that dichloroacetate (DCA), used clinically for the treatment of lactic acidosis, can function as an inhibitor of the PDK1/EGFR/EGFRvIII interaction. In addition to PDK1, the overexpression of EGFRvIII with EGFR is thought to be a hallmark for resistance to therapy^[73,74]^. This reverses the EGFR-mediated component of the Warburg effect by reducing lactate production in TMZ-resistant GBMs. Additionally, DCA preferentially binds EGFRvIII over EGFR, suggesting that DCA may become a useful alternative to treat chemotherapy-resistant GBM^[69]^.

It is known that low oxygen levels in the tumor stabilizes transcription factor HIF-1. HIF-1 modulates the expression of several glycolytic genes as well as playing a key regulatory role in apoptosis^[75-77]^. However, HIF-1α can be activated by PI3K/Akt leading to an increase in heat shock proteins 90 and 70, and stabilizing HIF-1α^[67]^. During the stabilization of HIF-1, HIF-1α and HIF-1β translocate to the nucleus and activate the vascular endothelial growth factor (VEGF) gene, increasing angiogenesis as a cellular response to hypoxia. GBM tumors often have increased expression of VEGF and VEGF receptor, VEGFR2, due to a hypoxic tumor microenvironment. This increased VEGF expression gives rise to the highly vascularized nature of GBM^[78,79]^. This provides a possible target to exploit therapeutically in TMZ-resistant GBMs^[80]^. HIF-1α has also been shown to stimulate the expression of CXCL12 and upregulate CXCR4^[81]^. Activation of CXCL12/CXCR4 plays a role in an autocrine/paracrine mechanism of GBM by stimulating tumor cell proliferation and peripheral invasiveness^[81]^. CXCL12/CXCR4 axis is a promising target for attenuating GBM TMZ resistance^[82]^.

A particular transcription factor targeted by Akt, FoxO3a, integrates cellular signals that control GSC differentiation and carcinogenesis, resulting in putative tumor suppressor function^[83,84]^. Its expression is usually tightly regulated by PI3K/Akt; in GBM however, where PI3K/Akt itself may be dysregulated, FoxO3a seems to function as a tumor enhancer rather than a tumor suppressor^[85]^. A recent study from Qian *et al*.^[85]^ examined 91 GBM samples and concluded that overexpression of FoxO3a correlates with tumor progression and is associated with poor prognosis. However, these findings are in contrast to a previous study conducted by Shi and coworkers^[84]^ where FoxO3a expression was determined in 70 human glioma specimens as well as 2 established GBM cell lines. The authors noted that lower expression of FoxO3a correlated with higher grade of the glioma and found that low expression of FoxO3a was associated with an overall poor patient outcome, and thus, high expression of FoxO3a was a favorable independent prognostic marker^[84]^. Given these conflicting results, the precise role of FoxO3a in GBM progression and patient prognosis remains elusive, as does its role in TMZ resistance, warranting further investigation before it can be effectively targeted as a potential therapeutic target.

**Figure 3:**
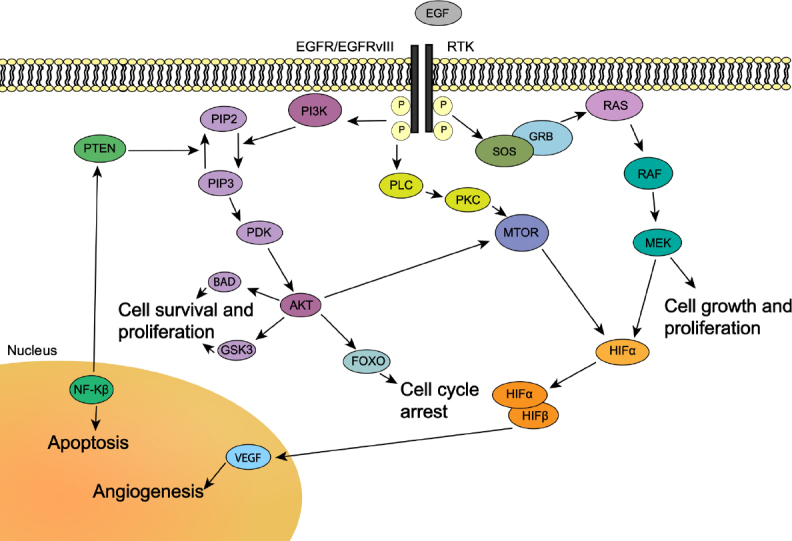
Molecular pathways associated with TMZ resistance. In the RTK pathway, activation of RTK results in downstream signaling controlling cell survival or apoptosis. Amplification of EGFR and inclusion of the mutant variant EGFRvIII, both RTKs, occurs in a majority of GBM. EGFRvIII has been shown to be a constitutively active mutant of EGFR, with ligand-independent activation resulting in RTK/RAS/PI3 dysregulation. Modified from Pearson *et al*.^[63]^. TMZ: temozolomide; RTKs: receptor tyrosine kinases; EGFR: epidermal growth factor receptor; EGFRvIII: epidermal growth factor receptor variant III

Yet another important target of Akt is NF-κB. Increases in NF-κB activity occur via phosphorylation and degradation of inhibitor of kappaB (IκB)^[86]^. NF-κB is identified as an oncogene in glioma and its overactivation is expectedly associated with poor prognosis. Several studies have described constitutively active NF-κB in response to stimuli, including cytokines in GBM cells. Through the activation of the RTK/PI3K/Akt pathway, NF-κB transcriptional activity is increased and activated NF-κB translocates to the nucleus where it initiates transcription of pro-survival genes^[87]^. NF-κB is implicated in TMZ resistance by studies hypothesizing that DNA damage caused by TMZ activates ataxia telangiectasia mutated (ATM) kinase which concurrently triggers MGMT repair and inappropriate activation of NF-κB^[87]^ [Figure 3].

Another Akt-mediated apoptotic target is the Bcl-2 family of proteins. The expression level of anti-apoptotic proteins (e.g., Bcl-2, Bcl-xl, Bcl-W and Mcl-1) as well as pro-apoptotic proteins (e.g., bax, box and bak) can determine the fate of cancer cells and in turn, the development of chemotherapeutic resistance^[88]^. In GBM stem cells, overexpression of anti-apoptotic proteins, and therefore an increase in Bcl-2/bak, plays a role in the escape from chemotherapy-induced death. This is mediated by the PI3K/Akt pathway, as it functions to inhibit the pro-apoptotic protein Bad^[89]^. Additionally, survivin, a member of the inhibitor of apoptosis family^[90]^, has been shown to block the effect of TMZ-induced apoptosis, conferring TMZ resistance. Specific inhibition of the survivin gene has also been shown to increase TMZ sensitivity^[91]^.

Together, the many downstream targets of the Akt pathway have cumulative effects that predispose to the development of resistance when they are mutated or perturbed by upstream dysregulation. Disrupting the proper activity and balance of cell survival and proliferation, angiogenesis, cell cycle arrest and apoptosis creates a cellular environment that is more likely to result in resistance to TMZ. These key molecular players should be kept in mind when designing targeted treatments or conducting molecular profiling of GBM tumors to avoid the development of resistance to TMZ.

### Wnt/β-catenin

**Figure 4:**
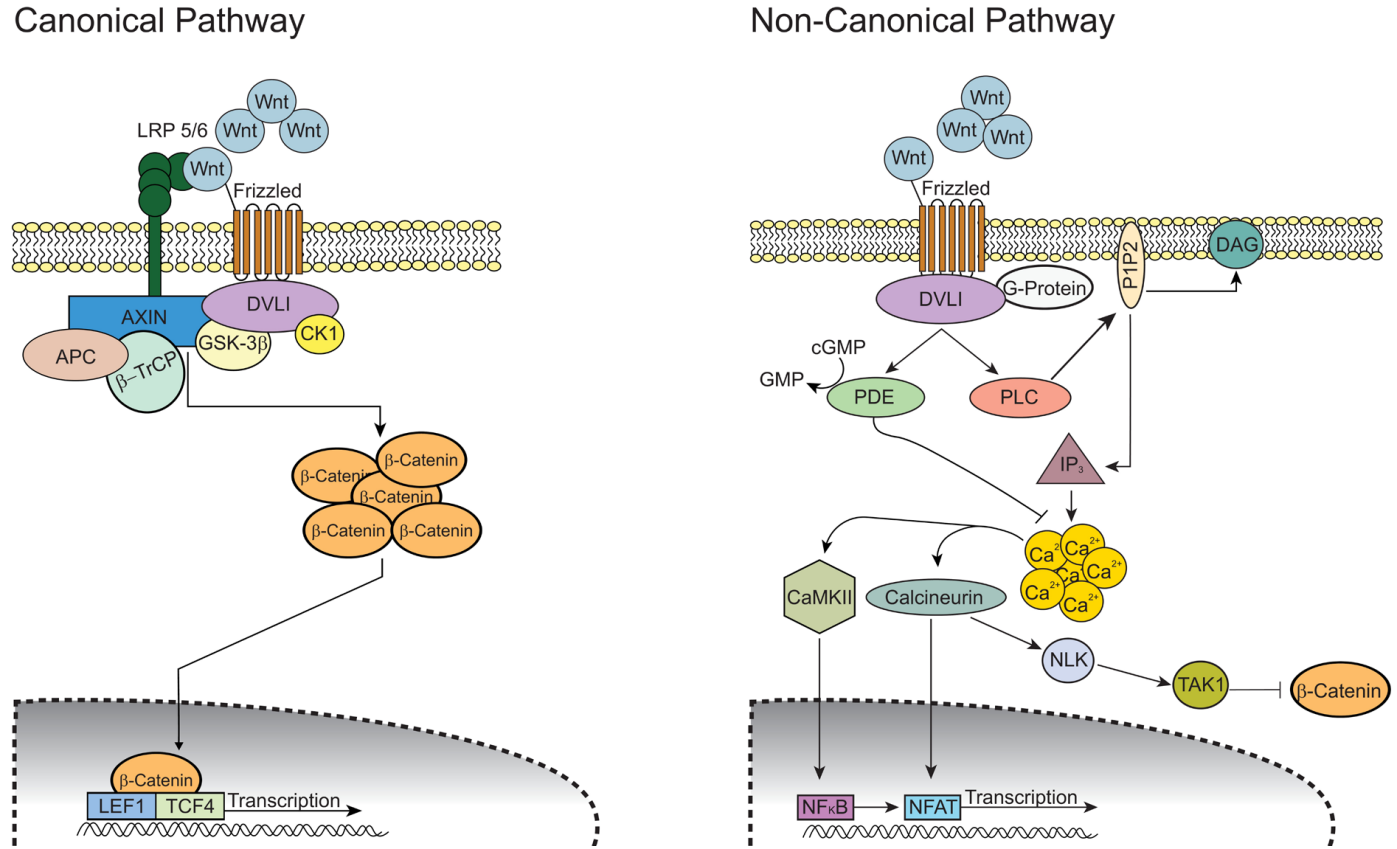
Wnt/β-catenin signaling pathways. In the canonical (β-catenin-dependent) pathway, Wnt ligand binds to its Frizzled receptor and co-receptor LRP 5/6. Upon activation, the signal is transduced and DVL1 gets activated which in turn inhibits GSK-3β and CK1 activity. This inhibition results in accumulation of intracellular β-catenin and its translocation into the nucleus. β-catenin acts as a transcriptional regulator along with LEF1 and TCF4. This transcriptional complex induces the expression of Wnt target genes that promote cell proliferation and differentiation. In the non-canonical (β-catenin-independent) pathway, Wnt ligands binds to its Frizzled receptor and triggers DLV1 to activate the cGMP-specific PDE and PLC. Activated PLC cleaves the membrane-bound PIP2 into IP3 and DAG. IP3 induces intracellular Ca^2+^ release from the endoplasmic reticulum. The resulting Ca^2+^ activates both CaMKII and calcineurin. CaMKII activates transcription factor NF-κB and calcineurin activates transcription factor NFAT. Cytoplasmic levels of β-catenin are kept low by downstream kinases of calcineurin NLK and TAK1. Modified from Tompa *et al*.^[92]^

The Wnt/β-catenin pathway plays a key role in the development of gliomas^[92]^. There are two main Wnt pathways: the canonical (β-catenin-dependent) and the non-canonical (β-catenin-independent) pathway [Figure 4]. Irregular signaling in both pathways contribute to the progression of GBM and chemoresistance. Ligands that are highly associated with the canonical pathway include Wnt1, Wnt3a and Wnt7a^[92]^. Riganti *et al*.^[93]^ showed that Wnt3a functions to induce stemness, is highly expressed in GBM tumors, and serves as an effective inducer of TMZ resistance in GBM. Furthermore, nuclear accumulation of β-catenin is indicative of activation of the canonical pathway^[94,95]^ and has been shown to play a role in TMZ resistance. One group has shown that FoxO3a facilitates the binding of β-catenin and nuclear import and thus promotes nuclear accumulation of β-catenin^[96]^. Aberrant activation of the canonical pathway has also been shown to play a role in the development of GSCs^[97]^. In addition, faulty regulation of the non-canonical pathway increases the tumorigenicity of GBM^[98]^.

Much like in other cancers, this pathway regulates self-renewal and differentiation in adult stem cells and influences stemness and cell behavior of tumor cells^[92]^. The activity of Wnt signaling is typically upregulated in GSCs compared to normal stem cells^[99]^. Wnt signaling also has an established role in the epithelial-mesenchymal transition (EMT) in gliomas^[100,101]^. This transition is related to enhancing cell motility and tumor spread and has also been associated with the resistance of GBM to current chemo- and radiotherapy^[102]^. Kahlert *et al*.^[103]^ showed that the modulation of Wnt signaling altered the expression of EMT activators. A more recent study revealed that endothelial cells (ECs) acquire transformation into mesenchymal-like cells in GBM leading to chemoresistance^[104]^. This group identified a c-Met-mediated axis that induced β-catenin phosphorylation at Ser^675^ and Wnt signaling activation, inducing multidrug resistance-associated protein-1 expression and leading to EC stemness-like activation and chemoresistance^[104]^. The authors also demonstrated that genetic ablation of β-catenin in ECs overcome GBM tumor resistance to TMZ chemotherapy *in vivo*. These findings showed a cell plasticity-based, microenvironment-dependent mechanism that controls tumor chemoresistance. Other modulators have been identified as key players in promoting GSC self-renewal and EMT and thereby promoting GBM growth and chemoresistance. These include SNAI2 from microRNA-203 (miR-203) downregulation^[105]^, FoxM1^[106]^, PLAGL2^[107]^, receptors FZDZ 2, 3 and 7^[108]^, and stem cell factor Wnt5a^[98]^. Furthermore, β-catenin has been shown to modulate EMT through upregulation of multiple target genes including ZEB1, Snail, Slug, Twist and several other transcription factors^[103,109]^, all of which play a role in promoting tumor growth. Conversely, one specific protein, secreted Frizzled-related protein 4, has been shown to be involved in the non-canonical pathway and actually works to induce differentiation of GSCs and increase TMZ sensitivity^[110]^.

Wnt signaling is also involved in the regulation of MGMT expression^[111]^. Given the well-established role Wnt signaling plays in gliomas, several therapeutic approaches have been investigated that target this signaling pathway, specifically those that modulate MGMT expression. Several Wnt inhibitors, such as salinomycin, celecoxib, and Wnt-C59, have shown restoration of TMZ sensitivity in resistant GBM cells. These small molecular inhibitors decrease the expression of MGMT in GSCs and potentiate TMZ sensitivity^[111]^.

One study further elucidated the potential role Wnt/β-catenin plays in glioma cells by demonstrating that TMZ treatment increased the activity from TOPflash reporter, (a Wnt-responsive reporter), enhanced the levels of pGSK-3β (S9), and reduced the levels of p-β-catenin with a concomitant increase in transcript and protein levels of Wnt targets in a concentration- and time-dependent manner^[112]^. While TMZ-treated cells did not show alteration in any of the Wnt ligands, PI3K inhibitor treatment repressed Akt activation and eliminated the TMZ-mediated induction of Wnt/β-catenin pathway. This group also showed that Wnt/β-catenin signaling activation by TMZ is independent of the ATM/Chk2 pathway, suggesting that activation of the Wnt/β-catenin pathway involves an ATM/Chk2-independent PI3K/Akt/GSK-3 cascade in TMZ-treated cells^[112]^.

Another protein, FERMT3, also known as kindlin-3, has been shown to play a critical role in the development and progression of GBM and chemoresistance to TMZ. FERMT3 is a cytosolic, adaptor protein and functions as an important activator and regulator of integrin functioning. One group determined that FERMT3 regulates glioma cell activity through integrin-mediated Wnt/β-catenin signaling; knockdown of this protein decreases GBM resistance to TMZ through downstream inhibition of β1-integrin, another key protein involved in GBM invasion and drug resistance^[113]^.

While research highlighting the integral role of microRNAs (miRNAs) in GBM is discussed in a later section, here we identify a few key miRNAs modulating the Wnt signaling pathway. For example, one study showed downregulation of miR-126-3p in TMZ-resistant GBM cells; higher levels of miR-126-3p were shown to inhibit Wnt signaling and enhance chemosensitivity^[114]^. Another study demonstrated that miR-129-5p acts as a tumor suppressor that modulates Wnt5a, a potent stem cell factor, and downstream factors such as NF-κB and JNK pathways. Downregulation of miR-129-5p promotes tumor cell proliferation and infiltration as well as TMZ resistance^[115]^. Other reports indicate that the upregulation of miR-21 corresponds with activated Wnt signaling and increased Bcl-2:Bax ratio resulting in GBM tumor growth^[116,117]^. A regulatory circuit including miR-125b/miR-20b and Wnt signaling has been shown to regulate both proneural and mesenchymal phenotypes of GBM^[118]^. In the proneural subtype, Wnt signaling is more active due to upregulation of miR-125b/miR-20b which in turn downregulates FZD6 and APC to maintain Wnt activation. FZD6 suppresses Wnt by activating the CaMKII-TAK1-NLK pathway, which activates STAT3 and NF-κB signaling. Overall, upregulation of miR-125b/miR-20b results in lack of Wnt attenuation and promotes cell growth of proneural GSCs^[118]^. Also, insulin-like growth factor 1 stimulates glioma proliferation by upregulating miR-513a-5p which acts to suppress expression of NEDD4L via PI3K signaling; this in turn prevents NEDD4L from inhibiting Wnt/β-catenin signaling and promotes GBM progression and chemoresistance^[119]^.

### JAK/STAT

The JAK/STAT signaling pathway is associated with stimulating glial stemness; and more specifically, activation of STAT3 has been demonstrated to affect the transition of GBM from the proneural subtype to the mesenchymal subtype^[120]^. STAT3 is activated upon phosphorylation followed by nuclear translocation, where STAT3 functions as a transcription factor that modulates the expression of a variety of oncogenic genes, including oncostatin M receptor, neurotensin, SOX2 and enhancer of zeste homolog 2 (EZH2)^[121-124]^. In addition, the interleukin 6/Janus kinase/STAT3 (IL-6/JAK/STAT3) pathway is involved in the pathogenesis of GBM. It has previously been reported that constitutively active STAT3 is frequently expressed in high-grade gliomas^[125]^. The dynamic interplay between the PI3K/AKT/NF-κB and JAK/STAT signaling pathway is illustrated by the activation of NF-κB, which leads to STAT3 expression^[126-128]^. Overactivation of STAT3 is also associated with the upregulation of MGMT expression and thus promotes TMZ desensitization and chemoresistance in GBM^[129]^. It has also been reported that STAT3 regulates the expression of miR-17, which modulates the MAP-ERK kinase pathway^[130]^.

In addition to promoting the infiltration of immune cells into the tumor^[131]^, IL-6 has also been reported to enhance STAT3 phosphorylation, which increases anti-apoptosis of tumor cells; specifically, overexpressed IL-6 has been identified as a marker of malignancy in GBM^[131]^. Wang *et al*.^[132]^ showed that inhibition of STAT3 dramatically decreased the IC50 of TMZ, increasing TMZ-induced apoptosis while upregulating Bcl-2 expression and downregulating Bax expression. Furthermore, they showed that inhibition of STAT3 increased TMZ-induced G_0_-G_1_ arrest and decreased cyclin D1 expression compared to TMZ alone. A more recent study pointed to histone 2A family members (H2AFs) as components of nucleosomes crucial for gene regulation in the host^[133]^. This group found that H2AFJ, but not other H2AFs, is commonly found and upregulated in mesenchymal-type GBM tumors, compared to its expression in normal tissues derived from GBM patients^[28]^. This study showed that H2AFJ knockdown dramatically suppressed IL-6 expression and NF-κB activity in GBM cells. Thus, H2AFJ is thought to be associated with the activation of TNF-α-NF-κB/IL-6-STAT3 signaling pathways. Snail family transcriptional repressor 1 (Snai1) has been documented as the key modulator of EMT, as well as in regulation of GBM tumor progression^[134]^. One study identified that Snail is upregulated in TMZ-resistant tumors^[135]^. The same authors showed that the increased secretion of IL-6 in resistant GBM promotes the transcription of Snail by targeting STAT3. This study revealed that the expression of Snail in GBM resistant cells is modulated by STAT3, which is stimulated by the over-secretion of IL-6^[135]^.

As for therapeutic approaches that target the STAT3 pathway, a potential method was recently identified and includes the use of salicylic acid-based inhibitor, SH-4-54, which was found to be toxic to TMZ-resistant GBM cells^[136]^. This group found that SH-4-54 leads to the activation of mitochondrial STAT3 (mitoSTAT3), negative regulation of mitochondrial-encoded genes, and abnormal oxidative phosphorylation.

## Autophagy in temozolomide resistance

Autophagy has a cytoprotective role in tumor cells and is noted for regulating metabolic homeostasis in various conditions such as hypoxia, chronic nutrient depletion, DNA damage and radiation^[137,138]^. In general, autophagy is a process by which cellular constituents are removed and targeted for destruction^[139]^. Furthermore, it is characterized by the segregation of damaged or unwanted cytoplasmic proteins and organelles, including mitochondria and endoplasmic reticula (ER), into autophagosomes that are ultimately tagged for lysosomal degradation^[139]^. This mechanism of eliminating damaged cellular contents protects cells from undergoing apoptosis^[140]^. This process is activated in tumor cells by chemotherapeutic agents and radiation^[141,142]^. Autophagy induced by TMZ treatment mostly functions as a survival and protective mechanism, and is considered as a mechanism of chemoresistance^[142]^.

### Molecular mechanisms of autophagy-mediated resistance

**Figure 5:**
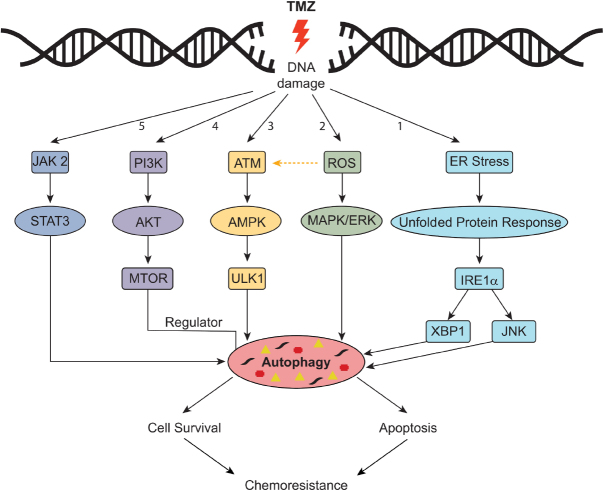
Mechanisms of resistance through TMZ-induced autophagy. Pathway #1: Endoplasmic reticulum (ER) stress triggers the unfolded protein response; downstream factors including IRE1 activate XBP1 and molecules downstream of JNK. Pathway #2: Accumulation of reactive oxygen species (ROS) results in activation of the MAP/ERK pathway. Pathway #3: Activation of ATM/AMPK pathway. Pathway #4: Activation of P13K/AKT pathway. Pathway #5. Activation of JAK2/STAT3 pathway. Modified from Hombach-Klonisch *et al*.^[145]^. TMZ: temozolomide

The role of autophagy has been largely controversial as it may lead to cancer death or cancer survival^[143,144]^. Several molecular mechanisms have been proposed for the activation of autophagy by chemotherapeutic agents [Figure 5]^[145]^. The accumulation of reactive oxygen species (ROS) leads to activation of the MAPK/ERK signaling pathway and stimulation of autophagy^[146]^. Moreover, activation of ATM/AMPK and inhibition of the PI3K/Akt/MTOR pathway has been shown to promote autophagy, as demonstrated by the treatment of GBM8901 cells with TMZ and carbazole alkaloid BC3EE2,9B^[147]^. Activation of the ATM/AMPK/ULK1 pathway has also been shown to activate autophagy in GBM cell models^[148,149]^. Under ER stress, IRE1 activates XBP1, ASK1 and molecules downstream of JNK that promote autophagy and apoptosis^[150]^; similar activation has also been noted in U87MG and GBM8401 cell lines exposed to TMZ^[146]^.

#### Early- and late-stage autophagy therapeutic modulators

Under certain cellular conditions, inhibition of autophagy can increase TMZ-induced apoptosis; treatment of these resistant cells with autophagy inhibitors or knockout of key autophagy genes including ATG5, ATG7, BECN1 or ATG4C have been shown to induce apoptosis^[151-153]^. Autophagy has also been shown to promote cell survival^[154]^ and avoid apoptosis by inhibiting caspase-8 activation^[155,156]^. The cytoprotective role of autophagy is further highlighted by two studies that showed that inhibition of autophagy combined with TMZ treatment at clinical therapeutic doses (≤ 100 μmol/L) produced increased levels of cell death^[157,158]^. Additionally, plant-derived compounds including resveratrol and ATM kinase inhibitors have been shown to inhibit autophagy and increase TMZ sensitivity as evidenced by decreased tumor volumes and increased survival times^[44,146]^. However, several therapeutics such as rapamycin inhibitors, thioridazine and momelotinib have been shown to enhance TMZ toxicity through induction of autophagic cell death in GBM tumors^[159-161]^, thus revealing the alternate role of autophagy in promoting apoptosis.

Several groups have identified autophagy-modulating agents that promote TMZ cytotoxicity at different stages. For example, 3-methyladenine exhibits decreased TMZ-induced cytotoxicity due to its target at early-stage autophagy inhibition^[143,162]^. Contrary to early-stage autophagy inhibitors, bafilomycin A1 inhibits TMZ-induced late-stage autophagy, increases the permeability of both mitochondrial and lysosomal membranes, and activates caspase-3, ultimately resulting in effective apoptotic cell death^[143]^. Another late-stage autophagy modulator includes chloroquine and its derivatives, which work to increase lysosomal pH and interrupt fusion of autophagosomes with lysosomes. This ultimately leads to an aberrant accumulation of vacuoles, inhibition of autophagic flux, and apoptosis^[163]^. Importantly, a subsequent phase I/II trial of hydroxychloroquine (HCQ) used in combination with radiation therapy and concurrent TMZ in newly-diagnosed GBM showed that at 600 mg/d HCQ (determined as the maximum tolerated dose), autophagy inhibition was not adequately achieved and that no significant improvements in overall survival were observed^[164]^. These data suggest that while autophagy plays a role in chemoresistance, the use of HCQ in this setting may not be advantageous.

The use of histone deacetylase (HDAC) inhibitors, including propylpentanoic acid and vorinostat, have previously been reported to enhance apoptotic and autophagic cell death^[165]^. More recently, a study looked into the effects of combining HDAC inhibitor suberoyl anilide hydoxamic acid (SAHA) with chloroquinoline to assess autophagic GBM cell apoptosis. Findings showed significant GBM cell death (tested in U87MG cells) resulting from elevated ROS levels and reduction in mitochondrial autophagy, both triggering apoptosis^[166]^. Modulation of autophagic flux potentiated SAHA-mediated apoptosis.

Harder *et al*.^[62]^ also identified an inhibitor of autophagic flux, PX-866, an irreversible pan-PI3K inhibitor, as a potential therapeutic target for TMZ-resistant GBM tumors. This blood-brain barrier penetrant was found to block autophagosome maturation in T986G cells^[62]^. The mechanism of inhibition has not been fully elucidated but is thought to work via inhibition of class III PI3K (VPS34), a key regulator of autophagosome synthesis^[167,168]^. Furthermore, this study provided evidence that TMZ-induced autophagy is a delayed response, thus suggesting the potential for sequential treatment (compared to concurrent treatment suggested for bafilomycin A1) with TMZ and PX-866 to block autophagic processes, enhance TMZ cytotoxicity and promote cell death.

As mentioned earlier, autophagy-mediated apoptosis (compared to cytoprotection) has also been an area of active research. Several groups have reported on thioridazine, an antipsychotic drug, which promotes the accumulation of LC3I/II and P62 (markers indicative of autophagy inhibition), compromises late-stage autophagy, and results in increased TMZ sensitivity^[159]^. A follow-up study further elucidated the mechanism by which thioridazine induces autophagy in GBM cells. The authors showed that thioridazine results in upregulation of AMPK activity and enhanced P62-mediated autophagy and apoptosis through Wnt/β-catenin signaling^[169]^.

Liu *et al*.^[161]^ demonstrated that mometinib, an ATP-competitive inhibitor of JAK-1 and JAK-2, increases autophagy and promotes apoptosis in TMZ-resistant GBM cells. This study showed that mometinib inhibits the phosphorylation of JAK2 and STAT3 in U251 xenograft mouse cells, ultimately blocking the JAK2/STAT3 pathway which leads to activation of autophagy. Furthermore, STAT3 activation is known to regulate downstream target anti-apoptotic genes Bcl-2 and Bcl-xL; therefore, inhibition of STAT3 effectively results in a decrease in the expression of these anti-apoptotic factors. These findings are consistent with previous studies that have shown the efficacy of Bcl-2 inhibitors in potentiating caspase-independent autophagic cell death in GBM tumors^[170-172]^.

Other autophagy-mediated apoptosis modulators, including Δ9-tetrahydrocannabinol^[173]^ and oncolytic adenovirus CRAd-Surivin-pk7^[174]^, have been shown to inhibit tumor growth in part when used in combination with TMZ. These results suggest that autophagy-associated apoptosis could potentially constitute a viable therapeutic approach to improve conventional GBM treatments.

#### Autophagy and endoplasmic reticulum stress

Despite increasing research in the field, the exact conditions under which autophagy either promotes apoptosis or maintains cellular homeostasis has not been fully determined. He and coworkers proposed that the level of ER stress (e.g., mild, moderate or severe) dictates whether autophagic pathways will promote apoptosis or activate protective mechanisms to maintain cellular homeostasis and avoid cell death^[140]^. ER stress inducers (e.g., nutrient deprivation, hypoxia, chemotherapeutic drugs or oxidative stress) can lead to an accumulation of aberrant proteins and activate the unfolded protein response (UPR), a key regulator and downstream target of autophagy^[140]^. Their model suggests that the effects of ER stress-induced autophagy on cell survival has both pro-survival and pro-death roles. Under mild to moderate ER stress, the UPR pathway will activate autophagic processes that promote removal of the damaged cellular constituents but evade apoptosis through inhibition of caspases. Conversely, under severe ER stress, autophagic processes will activate caspases to promote apoptosis. Several mitochondrial electron transport chain inhibitors as well as ER stress inhibitors have been explored given their inhibitory effects on autophagy; mediators including rotenone, oligomycin, sodium azide and ER stress inhibitor 4-phenylbutyrate have shown improvements in TMZ cytotoxicity by blocking autophagic processes^[146,175]^.

## Alternative mechanisms in temozolomide resistance

### Epigenetic modifications

Histone acetylation is an important epigenetic modification that modulates chromatin structure and regulates gene expression. Histone acetyltransferases add acetyl groups to lysine residues on histones to create open chromatin favoring transcriptional activation, while HDACs remove acetyl groups from lysine to create a compact chromatin structure favoring transcriptional silencing^[176]^. Importantly, HDACs have many targets beyond histones, including transcription factors, DNA repair enzymes, inflammatory mediators, signaling molecules, and structural proteins; therefore, HDAC activity can be thought of more broadly as a lysine deacetylase function^[177]^. The extensive effects of HDACs outside of epigenetic modification are supported by phylogenetic analyses showing HDAC evolution preceding that of histone proteins and the widespread cellular effects of altering HDAC activity^[178]^.

Mutations in HDAC enzymes are associated with tumor development, likely due to the resulting dysregulation of pathways involved in cell proliferation, cell cycle regulation and apoptosis and changes in the transcriptional status of oncogenes and tumor suppressors^[179]^. This observation has led to robust investigation of HDAC inhibitors as potential cancer treatments in recent years^[180,181]^. There are 18 unique HDAC enzymes that have been identified and classified in humans, and current developed HDAC inhibitors include both specific and pan-HDAC inhibitors^[177]^. HDAC inhibitors cause cancer cell death through cell cycle arrest, differentiation and apoptosis with increased efficacy when combined with other targeted treatments^[182,183]^. Three specific HDAC inhibitor drugs, vorinostat, romidepsin and valproic acid (an anticonvulsant drug with HDAC inhibitory effects) have been tested in clinical trials for GBM with many additional agents being investigated in preclinical studies^[184,185]^.

Several particular HDACs have been found to be upregulated in GBM including HDAC1, HDAC2, HDAC3, HDAC6, HDAC8 and HDAC9^[170,186,187]^. HDAC expression levels have also been found to correlate with tumor grade and survival, and overexpression has mechanistic linkages to tumor progression. For example, HDAC1 has been found to be upregulated in glioma cells and linked to proliferation and invasion through the activation of PI3K/Akt and MEK/Erk pathways^[188]^. Inhibition of overexpressed HDAC6 in GBM leads to decreased cell migration and tumor growth, thought to be mediated by inhibiting autophagy and activating antitumor immune responses^[189]^. Since many of the pathways targeted by HDACs are implicated in the development of resistance to TMZ therapy, HDAC inhibition may provide a way to circumvent TMZ resistance.

More direct evidence from recent studies suggests that HDAC inhibitors may help sensitize TMZ-resistant cells, making these drugs an attractive option for combined therapies^[185]^. At the most basic level, HDAC inhibition favors an acetylated, open chromatin state, giving TMZ better access to DNA. However, the elucidation of several unique mechanisms of HDAC regulation in TMZ-resistant GBM cells will provide new implications for treatment with selective HDAC inhibition. HDAC8-specific inhibition has been found to downregulate MGMT expression through an interaction with the proteasome receptor ADRM1, leading to increased cell damage, cell cycle arrest and cell death. However, this occurs only in TMZ-sensitive cells, suggesting that this mechanism may be disrupted in the context of TMZ resistance^[190]^. Selective inhibition of HDAC6 also increases TMZ sensitivity and induces apoptosis through upregulation of the MMR proteins MHS2 and MSH6, in addition to blockage of aberrant EGFR and p53 pathways^[191]^. A study by Li *et al*.^[192]^ found that a pharmacological inhibitor of HDAC1 and HDAC3 was able to overcome TMZ resistance via downregulation of NF-κB-regulated pro-survival genes. These findings support the multifactorial benefits of HDAC inhibitor therapy, in which multiple tumorigenic pathways can be targeted with a single drug, as well as demonstrating promise for the synergistic effects of chemotherapy and HDAC inhibitors in treating GBM without development of resistance.

### MicroRNAs

**Figure 6:**
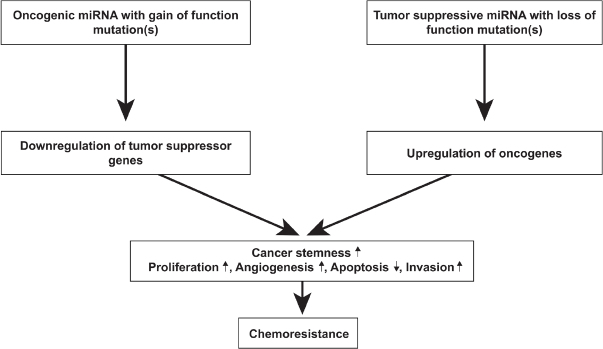
Role of miRNA in GBM tumorigenesis. MicroRNAs exert their effects by binding and interacting with their target mRNA, resulting in degradation or translational repression of the target protein. Due to their function, miRNAs effectively function as either oncogenic miRNA or tumor suppressive mRNA. miRNAs are oncogenic when their target proteins are tumor suppressors; conversely, miRNAs are tumor suppressive when their target proteins are oncogenic. The downregulation of tumor suppressor proteins and upregulation of oncogenic proteins can cause an increase in cancer stemness, promote cell proliferation, angiogenesis and invasion and inhibit apoptosis. Through these mechanisms, miRNAs play a key role in chemoresistance. Modified from Mizoguchi *et al*.^[197]^. GBM: glioblastoma

MicroRNAs (miRNAs; miRs) have been implicated in various biological processes underlying the phenomenon of therapeutic resistance^[193]^. miRNA expression profiles are largely diverse across patients, making it difficult to predict tumor response to treatments such as TMZ. miRNAs are non-coding RNAs that are endogenously expressed and regulate gene expression post-transcriptionally and are involved in processes including cell cycle regulation, proliferation, angiogenesis and GSC behavior^[193-195]^. miRNAs exhibit their effects by binding and interacting with target mRNA which results in degradation or translational repression of the target protein^[196]^. Some miRNAs are tumor suppressive while others are oncogenic; dysregulation via upregulation of oncogenic miRNAs or downregulation of tumor suppressive miRNAs can result in the activation of specific signaling pathways that result in GBM tumorigenesis and treatment resistance [Figure 6]^[197-200]^. One study identified miR-221 as upregulated in GBM and miR-128, miR-18a, miR-18b and miR-181c as downregulated in GBM^[193]^. Interestingly, this same group found that miR-21 was the most upregulated miRNA in GBM^[193]^. Several groups further investigated the mechanism by which miR-21 promotes chemoresistance; they showed that miR-21 overexpression promotes a decrease in the Bax/Bcl-2 ratio and decrease in caspase-3 activity, resulting in inhibition of TMZ-induced apoptosis^[201,202]^. Several miRNAs have been shown to regulate TMZ chemosensitivity; these include miR-128-1, miR-128-2, miR-130a, miR-181a, miR-221, miR-149, miR-125b, miR-31, miR-21, miR-222, miR-210, miR-195, miR-455-3p, miR-155-3p and miR-10a^[201-215]^. Chen *et al*.^[216]^ used bioinformatics analyses and immunoblotting to reveal that miR-155-3p targets Six1 which contributes to tumor proliferation and TMZ resistance. Additionally, several miRNAs involved in MGMT regulation have also been identified. Downregulation of miR-142-3p, miR-181d and miR-370-3p, and upregulation of miR-221/222, miR-370-3p, miR-409-3p, miR-603, miR-648 and miR-767-3p have been shown to inhibit MGMT suppression and promote chemoresistance in GBM cells^[213,217-224]^.

Modulating dysregulated miRNA expression profiles in TMZ-resistant GBM cells exposes a novel therapeutic target to improve the effects of conventional therapies. Ujifuku *et al*.^[116]^ demonstrated that miR-195 inhibition enhanced TMZ sensitivity and promoted apoptosis. As mentioned earlier, overexpression of miR-21 is noted in TMZ-resistant GBM, and miR-21 inhibitors such as paclitaxel, doxorubicin, sunitinib and VM-26 have been shown to increase the effectiveness of TMZ treatment^[210,225-229]^. Results of these miR-21 inhibitors, tested in cell lines U87 human and F98 rat glioma cells, represents a promising therapeutic approach towards GBM^[228]^. Wang *et al*.^[230]^ demonstrated that the introduction of miR-130-3p greatly increases TMZ sensitivity by inhibiting the expression of specificity protein 1 (Sp1), a protein involved in cell proliferation and chemoresistance^[230-232]^. Yang *et al*.^[233]^ showed that miR-204 expression was downregulated in U251MG resistant cells; they concluded that the expression of miR-204 can reverse TMZ resistance and inhibit cancer-initiating cells via degradation of fibroblast activation protein a in GBM. As discussed, specific miRNAs are involved in signaling pathways that confer resistance to TMZ. As previously mentioned, constitutively active STAT3 confers chemoresistance in GBM. One group found that forced expression of miR-31 reduced tumor growth and induced mitochondrial apoptosis through an increase in caspase-3 and caspase-9 activity^[203]^. There are also several miRNAs that activate autophagic processes. In particular, miR-17 is often upregulated in GBM tumors and specifically targets ATG7, a key regulator of autophagy, and promotes autophagy-induced cell protection and growth^[234,235]^. Research has shown that decreased expression of miR-17 results in an increase in ATG7 and subsequently decreases cell growth^[235]^, exposing a potential therapeutic approach in patients with overexpressed miR-17. New miRNAs involved in GBM are continuously being discovered and explored as potential therapeutic approaches to be used in conjunction with chemotherapy and/or radiation.

### Extracellular vesicles

miRNAs as well as other small cellular components such as enzymes, long non-coding RNAs (lncRNA), drug efflux pumps and extrachromosomal circular DNA play an additional role in resistance through their ability to be encapsulated and spread in extracellular vesicles (EVs). EVs are lipid bilayer vesicles that include both exosomes and microvesicles depending on size, exosomes being ~30-120 nm and microvesicles generally > 120 nm^[236]^. In GBM, EVs have been found to carry well-known tumor propagating factors such as EGFRvIII, SOX-2 and activators of the PI3K/Akt, PTEN, Erk and STAT3 pathways^[237,238]^. EVs also have the capacity to transfer resistance-mediating factors intercellularly, making them an important albeit unconventional mechanism of resistance^[237,239]^. For example, Zeng *et al*.^[240]^ showed that loss of miR-151a drives acquisition of TMZ resistance, and exosomes transferred from a population of resistant cells to a population of TMZ-sensitive cells were able to induce TMZ-resistance in a miR-151a loss-dependent manner.

EVs have been implicated in the overall progression of GBM disease and remodeling of the tumor microenvironment^[241,242]^. Thus, they may also specifically contribute to acquired TMZ resistance through transfer of resistance-mediating EVs to otherwise therapy sensitive cells or even non-cancerous cells. This has been found to be true in the case of lncSBF2-AS1-enriched exosomes that are able to promote TMZ-resistance. Zhang *et al*.^[241]^ found that lncSBF2-AS1 functions as a competing endogenous RNA of miR-151a-3p, disinhibiting its target XRCC4 and resulting in increased DNA repair and TMZ resistance. Another hallmark EV study showed that gene fusion elements play a role in EV-mediated resistance. EVs harboring PTPRZ1-MET fusions, a known driver mutation in GBM, altered gene expression in recipient cells, induced EMT transition and cell migration, and conferred TMZ resistance^[243]^. It has also been found that hypoxic stress induces changes in EV content, tying in yet another previous mechanism of TMZ resistance. Hypoxic exosomes have been shown to trigger gene expression changes in recipient cells such as upregulation of small nucleolar RNA C/D box 1116-21 (SNORD116-21) and downregulation of potassium voltage-gated channel subfamily member 3 (KCNJ3)^[244]^.

Furthermore, once EVs exit the cell, they may also enter the bloodstream, which has led to the investigation of EVs as potential cancer biomarkers and the possibility of liquid biopsy^[236]^. In the case of TMZ resistance and GBM, this presents the opportunity to screen for known resistance markers and use EVs in the prediction of therapy response. A previous study by Akers *et al*.^[245]^ showed that miR-21 can be used to reliably distinguish exosomes in CSF from GBM *vs*. non-oncological patients from which it can be extrapolated that the same process could also be used for miRNAs found to have specific roles in TMZ resistance. This has in fact been attempted in the form of microfluidic chip-based analysis of MGMT and APNG expression over time in seven GBM patients. Exosome samples taken from blood were able to show fluctuations in MGMT and APNG levels throughout treatment that may be suggestive of resistance development^[246]^. This increased understanding and utilization of EVs in diagnosis is representative of the many advancements in the field of TMZ resistance and a movement towards more novel and creative solutions to glioblastoma treatment^[247]^.

## Conclusion

**Figure 7:**
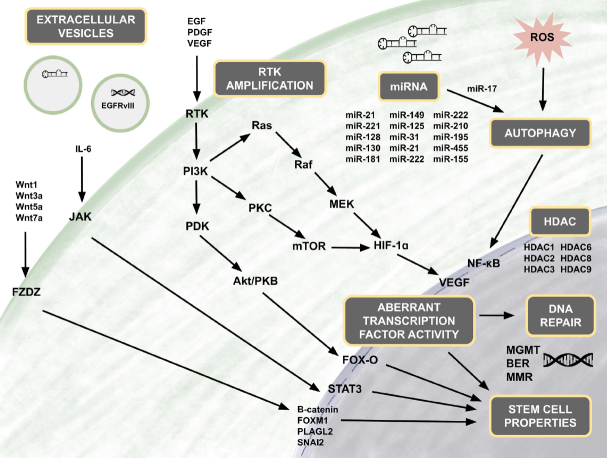
Overview of mechanisms involved in TMZ resistance in GBM. The molecular mechanisms contributing to TMZ resistance in GBM are nuanced and extensive yet fall primarily into the fundamental categories of DNA repair, cell stemness, HDAC activity, aberrant receptor and transcription factor activity, autophagy, activation and suppression by various miRNAs, and intercellular transport of oncogenic factors via extracellular vesicles. There is significant interplay between all of these categories, and often a mutation or overactivation in one category will prompt or amplify additional resistance pathways. For this reason, successful combating of TMZ resistance will require a multifaceted approach that prevents compensation through alternative resistance mechanisms. GBM: glioblastoma; TMZ: temozolomide; HDACs: histone deacetylases

TMZ resistance remains a major limitation in the treatment of GBM and contributes to the dismal prognosis. Development of TMZ resistance involves a complex interplay of numerous molecular mechanisms (summarized in Figure 7). A deeper understanding of the dysregulated pathways used by GBM cells will likely translate into the development of novel and more effective therapeutic approaches to overcome TMZ resistance and ultimately improve patient outcomes.

## Declarations

### Copyright

© The Author(s) 2021.

## References

[1] Ostrom QT, Cioffi G, Gittleman H (2019). CBTRUS statistical report: primary brain and other central nervous system tumors diagnosed in the United States in 2012-2016.. Neuro Oncol.

[2] Krex D, Klink B, Hartmann C (2007). Long-term survival with glioblastoma multiforme.. Brain.

[3] Johnson DR, O’Neill BP (2012). Glioblastoma survival in the United States before and during the temozolomide era.. J Neurooncol.

[4] Delgado-Lopez PD, Corrales-Garcia EM (2016). Survival in glioblastoma: a review on the impact of treatment modalities.. Clin Transl Oncol.

[5] Stupp R, Brada M, van den Bent MJ (2014). High-grade glioma: ESMO clinical practice guidelines for diagnosis, treatment and follow-up.. Ann Oncol..

[6] Carter TC, Medina-Flores R, Lawler BE (2018). Glioblastoma treatment with temozolomide and bevacizumab and overall survival in a rural tertiary healthcare practice.. Biomed Res Int.

[7] Seystahl K, Hentschel B, Loew S (2020). Bevacizumab versus alkylating chemotherapy in recurrent glioblastoma.. J Cancer Res Clin Oncol.

[8] Gerstner ER, Emblem KE, Chang K (2020). Bevacizumab reduces permeability and concurrent temozolomide delivery in a subset of patients with recurrent glioblastoma.. Clin Cancer Res.

[9] Champeaux C, Weller J (2020). Implantation of carmustine wafers (Gliadel ®) for high-grade glioma treatment. A 9-year nationwide retrospective study.. J Neurooncol.

[10] Ashby LS, Smith KA, Stea B (2016). Gliadel wafer implantation combined with standard radiotherapy and concurrent followed by adjuvant temozolomide for treatment of newly diagnosed high-grade glioma: a systematic literature review.. World J Surg Oncol.

[11] Mittal S, Klinger NV, Michelhaugh SK (2018). Alternating electric tumor treating fields for treatment of glioblastoma: rationale, preclinical, and clinical studies.. J Neurosurg.

[12] Kinzel A, Ambrogi M, Varshaver M, Kirson ED (2019). Tumor treating fields for glioblastoma treatment: patient satisfaction and compliance with the second-generation optune((R)) System.. Clin Med Insights Oncol.

[13] Guberina N, Pottgen C, Kebir S (2020). Combined radiotherapy and concurrent tumor treating fields (TTFields) for glioblastoma: dosimetric consequences on non-coplanar IMRT as initial results from a phase I trial.. Radiat Oncol.

[14] Stupp R, Taillibert S, Kanner A (2017). Effect of tumor-treating fields plus maintenance temozolomide vs maintenance temozolomide alone on survival in patients with glioblastoma: a randomized clinical trial.. JAMA.

[15] Fabian D, Guillermo Prieto Eibl MDP, Alnahhas I (2019). Treatment of glioblastoma (GBM) with the addition of tumor-treating fields (TTF): a review.. Cancers (Basel).

[16] Louis DN, Perry A, Reifenberger G (2016). The 2016 World Health Organization classification of tumors of the central nervous system: a summary.. Acta Neuropathol.

[17] Yang P, Zhang W, Wang Y (2015). IDH mutation and MGMT promoter methylation in glioblastoma: results of a prospective registry.. Oncotarget.

[18] Le Rhun E, Preusser M, Roth P (2019). Molecular targeted therapy of glioblastoma.. Cancer Treat Rev.

[19] Weller M, Stupp R, Reifenberger G (2010). MGMT promoter methylation in malignant gliomas: ready for personalized medicine?. Nat Rev Neurol.

[20] Hegi ME, Diserens AC, Gorlia T (2005). MGMT gene silencing and benefit from temozolomide in glioblastoma.. N Engl J Med.

[21] Chen R, Smith-Cohn M, Cohen AL, Colman H (2017). Glioma subclassifications and their clinical significance.. Neurotherapeutics.

[22] Arora A, Somasundaram K (2019). Glioblastoma vs temozolomide: can the red queen race be won?. Cancer Biol Ther.

[23] Mohammad SN, Hopfinger AJ (1980). Chemical reactivity of a methyldiazonium ion with nucleophilic centers of DNA bases.. J Theor Biol.

[24] Friedman HS, Kerby T, Calvert H (2000). Temozolomide and treatment of malignant glioma.. Clin Cancer Res.

[25] Stupp R, Mason WP, van den Bent MJ (2005). Radiotherapy plus concomitant and adjuvant temozolomide for glioblastoma.. N Engl J Med.

[26] Lee SY (2016). Temozolomide resistance in glioblastoma multiforme.. Genes Dis.

[27] Wick W, Platten M (2014). Understanding and targeting alkylator resistance in glioblastoma.. Cancer Discov.

[28] Xie Q, Mittal S, Berens ME (2014). Targeting adaptive glioblastoma: an overview of proliferation and invasion.. Neuro Oncol.

[29] Ignatova TN, Kukekov VG, Laywell ED (2002). Human cortical glial tumors contain neural stem-like cells expressing astroglial and neuronal markers in vitro.. Glia.

[30] Chen J, Li Y, Yu TS (2012). A restricted cell population propagates glioblastoma growth after chemotherapy.. Nature.

[31] Bao S, Wu Q, McLendon RE (2006). Glioma stem cells promote radioresistance by preferential activation of the DNA damage response.. Nature.

[32] Hassn Mesrati M, Behrooz AB, Abuhamad AY, Syahir A (2020). Understanding Glioblastoma Biomarkers: Knocking a Mountain with a Hammer.. Cells.

[33] Kim H, Zheng S, Amini SS (2015). Whole-genome and multisector exome sequencing of primary and post-treatment glioblastoma reveals patterns of tumor evolution.. Genome Res.

[34] Orzan F, De Bacco F, Crisafulli G (2017). Genetic evolution of glioblastoma stem-like cells from primary to recurrent tumor.. Stem Cells.

[35] Garnier D, Meehan B, Kislinger T (2018). Divergent evolution of temozolomide resistance in glioblastoma stem cells is reflected in extracellular vesicles and coupled with radiosensitization.. Neuro Oncol.

[36] Lan X, Jorg DJ, Cavalli FMG (2017). Fate mapping of human glioblastoma reveals an invariant stem cell hierarchy.. Nature.

[37] Cordner R, Black KL, Wheeler CJ (2013). Exploitation of adaptive evolution in glioma treatment.. CNS Oncol.

[38] Feldheim J, Kessler AF, Monoranu CM (2019). Changes of O(6)-methylguanine DNA methyltransferase (MGMT) promoter methylation in glioblastoma relapse-a meta-analysis type literature review.. Cancers (Basel).

[39] Rivera AL, Pelloski CE, Gilbert MR (2010). MGMT promoter methylation is predictive of response to radiotherapy and prognostic in the absence of adjuvant alkylating chemotherapy for glioblastoma.. Neuro Oncol.

[40] Park CK, Kim JE, Kim JY (2012). The changes in MGMT promoter methylation status in initial and recurrent glioblastomas.. Transl Oncol.

[41] Herrlinger U, Tzaridis T, Mack F (2019). Lomustine-temozolomide combination therapy versus standard temozolomide therapy in patients with newly diagnosed glioblastoma with methylated MGMT promoter (CeTeG/NOA-09): a randomised, open-label, phase 3 trial.. Lancet.

[42] Tang JB, Svilar D, Trivedi RN (2011). N-methylpurine DNA glycosylase and DNA polymerase beta modulate BER inhibitor potentiation of glioma cells to temozolomide.. Neuro Oncol.

[43] Trivedi RN, Almeida KH, Fornsaglio JL, Schamus S, Sobol RW (2005). The role of base excision repair in the sensitivity and resistance to temozolomide-mediated cell death.. Cancer Res.

[44] Agnihotri S, Burrell K, Buczkowicz P (2014). ATM regulates 3-methylpurine-DNA glycosylase and promotes therapeutic resistance to alkylating agents.. Cancer Discov.

[45] Thanasupawat T, Natarajan S, Rommel A, Glogowska A, Bergen H (2017). Dovitinib enhances temozolomide efficacy in glioblastoma cells.. Mol Oncol.

[46] Parsons JL, Dianova II, Allinson SL, Dianov GL (2005). Poly(ADP-ribose) polymerase-1 protects excessive DNA strand breaks from deterioration during repair in human cell extracts.. FEBS J.

[47] Woodhouse BC, Dianova II, Parsons JL, Dianov GL (2008). Poly(ADP-ribose) polymerase-1 modulates DNA repair capacity and prevents formation of DNA double strand breaks.. DNA Repair (Amst).

[48] Nakada M, Furuta T, Hayashi Y, Minamoto T, Hamada J (2012). The strategy for enhancing temozolomide against malignant glioma.. Front Oncol.

[49] Atkins RJ, Ng W, Stylli SS, Hovens CM, Kaye AH (2015). Repair mechanisms help glioblastoma resist treatment.. J Clin Neurosci.

[50] Gil Del Alcazar CR, Todorova PK, Habib AA, Mukherjee B, Burma S (2016). Augmented HR repair mediates acquired temozolomide resistance in glioblastoma.. Mol Cancer Res.

[51] Messaoudi K, Clavreul A, Lagarce F (2015). Toward an effective strategy in glioblastoma treatment. Part I: resistance mechanisms and strategies to overcome resistance of glioblastoma to temozolomide.. Drug Discov Today.

[52] Erasimus H, Gobin M, Niclou S, Van Dyck E (2016). DNA repair mechanisms and their clinical impact in glioblastoma.. Mutat Res Rev Mutat Res.

[53] Yoshimoto K, Mizoguchi M, Hata N (2012). Complex DNA repair pathways as possible therapeutic targets to overcome temozolomide resistance in glioblastoma.. Front Oncol.

[54] Zhang J, Stevens MF, Bradshaw TD (2012). Temozolomide: mechanisms of action, repair and resistance.. Curr Mol Pharmacol.

[55] Touat M, Li YY, Boynton AN (2020). Mechanisms and therapeutic implications of hypermutation in gliomas.. Nature.

[56] Perazzoli G, Prados J, Ortiz R (2015). Temozolomide resistance in glioblastoma cell lines: implication of MGMT, MMR, P-Glycoprotein and CD133 expression.. PLoS One.

[57] Stark AM, Doukas A, Hugo HH (2015). Expression of DNA mismatch repair proteins MLH1, MSH2, and MSH6 in recurrent glioblastoma.. Neurol Res.

[58] Indraccolo S, Lombardi G, Fassan M (2019). Genetic, epigenetic, and immunologic profiling of MMR-deficient relapsed glioblastoma.. Clin Cancer Res.

[59] Nagel ZD, Kitange GJ, Gupta SK (2017). DNA repair capacity in multiple pathways predicts chemoresistance in glioblastoma multiforme.. Cancer Res.

[60] Struve N, Binder ZA, Stead LF (2020). EGFRvIII upregulates DNA mismatch repair resulting in increased temozolomide sensitivity of MGMT promoter methylated glioblastoma.. Oncogene.

[61] Guo G, Sun Y, Hong R (2020). IKBKE enhances TMZ-chemoresistance through upregulation of MGMT expression in glioblastoma.. Clin Transl Oncol.

[62] Harder BG, Peng S, Sereduk CP (2019). Inhibition of phosphatidylinositol 3-kinase by PX-866 suppresses temozolomide-induced autophagy and promotes apoptosis in glioblastoma cells.. Mol Med.

[63] Pearson JRD, Regad T (2017). Targeting cellular pathways in glioblastoma multiforme.. Signal Transduct Target Ther.

[64] Network CGAR (2008). Comprehensive genomic characterization defines human glioblastoma genes and core pathways.. Nature.

[65] Westphal M, Maire CL, Lamszus K (2017). EGFR as a target for glioblastoma treatment: an unfulfilled promise.. CNS Drugs.

[66] Taylor TE, Furnari FB, Cavenee WK (2012). Targeting EGFR for treatment of glioblastoma: molecular basis to overcome resistance.. Curr Cancer Drug Targets.

[67] Zhou J, Schmid T, Frank R, Brüne B (2004). PI3K/Akt is required for heat shock proteins to protect hypoxia-inducible factor 1alpha from pVHL-independent degradation.. J Biol Chem.

[68] Zheng HC (2017). The molecular mechanisms of chemoresistance in cancers.. Oncotarget.

[69] Velpula KK, Guda MR, Sahu K (2017). Metabolic targeting of EGFRvIII/PDK1 axis in temozolomide resistant glioblastoma.. Oncotarget.

[70] Hitosugi T, Fan J, Chung TW (2011). Tyrosine phosphorylation of mitochondrial pyruvate dehydrogenase kinase 1 is important for cancer metabolism.. Mol Cell.

[71] Velpula KK, Bhasin A, Asuthkar S, Tsung AJ (2013). Combined targeting of PDK1 and EGFR triggers regression of glioblastoma by reversing the Warburg effect.. Cancer Res.

[72] Velpula KK, Tsung AJ (2014). PDK1: a new therapeutic target for glioblastoma?. CNS Oncol.

[73] Azuaje F, Tiemann K, Niclou SP (2015). Therapeutic control and resistance of the EGFR-driven signaling network in glioblastoma.. Cell Commun Signal.

[74] Wang H, Xu T, Jiang Y (2015). The challenges and the promise of molecular targeted therapy in malignant gliomas.. Neoplasia.

[75] Franovic A, Gunaratnam L, Smith K (2007). Translational up-regulation of the EGFR by tumor hypoxia provides a nonmutational explanation for its overexpression in human cancer.. Proc Natl Acad Sci U S A.

[76] Muñoz-Pinedo C, El Mjiyad N, Ricci JE (2012). Cancer metabolism: current perspectives and future directions.. Cell Death Dis.

[77] Plathow C, Weber WA (2008). Tumor cell metabolism imaging.. J Nucl Med..

[78] Steiner HH, Karcher S, Mueller MM (2004). Autocrine pathways of the vascular endothelial growth factor (VEGF) in glioblastoma multiforme: clinical relevance of radiation-induced increase of VEGF levels.. J Neurooncol.

[79] Joensuu H, Puputti M, Sihto H, Tynninen O, Nupponen NN (2005). Amplification of genes encoding KIT, PDGFRalpha and VEGFR2 receptor tyrosine kinases is frequent in glioblastoma multiforme.. J Pathol.

[80] Weathers SP, de Groot J (2015). VEGF manipulation in glioblastoma.. Oncology (Williston Park).

[81] Wurth R, Bajetto A, Harrison JK, Barbieri F, Florio T (2014). CXCL12 modulation of CXCR4 and CXCR7 activity in human glioblastoma stem-like cells and regulation of the tumor microenvironment.. Front Cell Neurosci.

[82] Wang S, Chen C, Li J (2020). The CXCL12/CXCR4 axis confers temozolomide resistance to human glioblastoma cells via up-regulation of FOXM1.. J Neurol Sci.

[83] Sunayama J, Sato A, Matsuda K (2011). FoxO3a functions as a key integrator of cellular signals that control glioblastoma stem-like cell differentiation and tumorigenicity.. Stem Cells.

[84] Shi J, Zhang L, Shen A (2010). Clinical and biological significance of forkhead class box O 3a expression in glioma: mediation of glioma malignancy by transcriptional regulation of p27kip1.. J Neurooncol.

[85] Qian Z, Ren L, Wu D (2017). Overexpression of FoxO3a is associated with glioblastoma progression and predicts poor patient prognosis.. Int J Cancer.

[86] Bai D, Ueno L, Vogt PK (2009). Akt-mediated regulation of NFkappaB and the essentialness of NFkappaB for the oncogenicity of PI3K and Akt.. Int J Cancer.

[87] Nogueira L, Ruiz-Ontanon P, Vazquez-Barquero A, Moris F, Fernandez-Luna JL (2011). The NFkappaB pathway: a therapeutic target in glioblastoma.. Oncotarget.

[88] Fu Z, Tindall DJ (2008). FOXOs, cancer and regulation of apoptosis.. Oncogene.

[89] Zhang X, Tang N, Hadden TJ, Rishi AK (2011). Akt, FoxO and regulation of apoptosis.. Biochim Biophys Acta.

[90] Salvesen GS, Duckett CS (2002). IAP proteins: blocking the road to death’s door.. Nat Rev Mol Cell Biol.

[91] Song Z, Pan Y, Ling G (2017). Escape of U251 glioma cells from temozolomide-induced senescence was modulated by CDK1/survivin signaling.. Am J Transl Res.

[92] Tompa M, Kalovits F, Nagy A, Kalman B (2018). Contribution of the Wnt pathway to defining biology of glioblastoma.. Neuromolecular Med.

[93] Riganti C, Salaroglio IC, Caldera V (2013). Temozolomide downregulates P-glycoprotein expression in glioblastoma stem cells by interfering with the Wnt3a/glycogen synthase-3 kinase/beta-catenin pathway.. Neuro Oncol.

[94] Lan F, Pan Q, Yu H, Yue X (2015). Sulforaphane enhances temozolomide-induced apoptosis because of down-regulation of miR-21 via Wnt/beta-catenin signaling in glioblastoma.. J Neurochem.

[95] Lu J, Zhang F, Zhao D (2008). ATRA-inhibited proliferation in glioma cells is associated with subcellular redistribution of beta-catenin via up-regulation of Axin.. J Neurooncol.

[96] Xu K, Zhang Z, Pei H (2017). FoxO3a induces temozolomide resistance in glioblastoma cells via the regulation of beta-catenin nuclear accumulation.. Oncol Rep.

[97] Morris LG, Kaufman AM, Gong Y (2013). Recurrent somatic mutation of FAT1 in multiple human cancers leads to aberrant Wnt activation.. Nat Genet.

[98] Kamino M, Kishida M, Kibe T (2011). Wnt-5a signaling is correlated with infiltrative activity in human glioma by inducing cellular migration and MMP-2.. Cancer Sci.

[99] Skoda J, Hermanova M, Loja T (2016). Co-expression of cancer stem cell markers corresponds to a pro-tumorigenic expression profile in pancreatic adenocarcinoma.. PLoS One.

[100] Fu Y, Zheng S, An N (2011). beta-catenin as a potential key target for tumor suppression.. Int J Cancer.

[101] Valenta T, Hausmann G, Basler K (2012). The many faces and functions of beta-catenin.. EMBO J.

[102] Zhang M, Atkinson RL, Rosen JM (2010). Selective targeting of radiation-resistant tumor-initiating cells.. Proc Natl Acad Sci U S A.

[103] Kahlert UD, Maciaczyk D, Doostkam S, Orr BA, Simons B (2012). Activation of canonical WNT/beta-catenin signaling enhances in vitro motility of glioblastoma cells by activation of ZEB1 and other activators of epithelial-to-mesenchymal transition.. Cancer Lett.

[104] Huang M, Zhang D, Wu JY (2020). Wnt-mediated endothelial transformation into mesenchymal stem cell-like cells induces chemoresistance in glioblastoma.. Sci Transl Med.

[105] Liao H, Bai Y, Qiu S (2015). MiR-203 downregulation is responsible for chemoresistance in human glioblastoma by promoting epithelial-mesenchymal transition via SNAI2.. Oncotarget.

[106] Zhang N, Wei P, Gong A (2011). FoxM1 promotes beta-catenin nuclear localization and controls Wnt target-gene expression and glioma tumorigenesis.. Cancer Cell.

[107] Zheng H, Ying H, Wiedemeyer R (2010). PLAGL2 regulates Wnt signaling to impede differentiation in neural stem cells and gliomas.. Cancer Cell.

[108] Sandberg CJ, Altschuler G, Jeong J (2013). Comparison of glioma stem cells to neural stem cells from the adult human brain identifies dysregulated Wnt- signaling and a fingerprint associated with clinical outcome.. Exp Cell Res.

[109] Garg M (2013). Epithelial-mesenchymal transition - activating transcription factors - multifunctional regulators in cancer.. World J Stem Cells.

[110] Bhuvanalakshmi G, Arfuso F, Millward M, Dharmarajan A, Warrier S (2015). Secreted frizzled-related protein 4 inhibits glioma stem-like cells by reversing epithelial to mesenchymal transition, inducing apoptosis and decreasing cancer stem cell properties.. PLoS One.

[111] Wickstrom M, Dyberg C, Milosevic J (2015). Wnt/beta-catenin pathway regulates MGMT gene expression in cancer and inhibition of Wnt signalling prevents chemoresistance.. Nat Commun.

[112] Tomar VS, Patil V, Somasundaram K (2020). Temozolomide induces activation of Wnt/beta-catenin signaling in glioma cells via PI3K/Akt pathway: implications in glioma therapy.. Cell Biol Toxicol.

[113] Lu C, Cui C, Liu B (2017). FERMT3 contributes to glioblastoma cell proliferation and chemoresistance to temozolomide through integrin mediated Wnt signaling.. Neurosci Lett.

[114] Luo W, Yan D, Song Z (2019). miR-126-3p sensitizes glioblastoma cells to temozolomide by inactivating Wnt/beta-catenin signaling via targeting SOX2.. Life Sci.

[115] Zeng A, Yin J, Li Y (2018). miR-129-5p targets Wnt5a to block PKC/ERK/NF-kappaB and JNK pathways in glioblastoma.. Cell Death Dis.

[116] Ujifuku K, Mitsutake N, Takakura S (2010). miR-195, miR-455-3p and miR-10a (*) are implicated in acquired temozolomide resistance in glioblastoma multiforme cells.. Cancer Lett.

[117] Zhang KL, Han L, Chen LY (2014). Blockage of a miR-21/EGFR regulatory feedback loop augments anti-EGFR therapy in glioblastomas.. Cancer Lett.

[118] Huang T, Alvarez AA, Pangeni RP (2016). A regulatory circuit of miR-125b/miR-20b and Wnt signalling controls glioblastoma phenotypes through FZD6-modulated pathways.. Nat Commun.

[119] Chen KC, Chen PH, Ho KH (2019). IGF-1-enhanced miR-513a-5p signaling desensitizes glioma cells to temozolomide by targeting the NEDD4L-inhibited Wnt/beta-catenin pathway.. PLoS One.

[120] Segerman A, Niklasson M, Haglund C (2016). Clonal variation in drug and radiation response among glioma-initiating cells is linked to proneural-mesenchymal transition.. Cell Rep.

[121] Jahani-Asl A, Yin H, Soleimani VD (2016). Control of glioblastoma tumorigenesis by feed-forward cytokine signaling.. Nat Neurosci.

[122] Kim E, Kim M, Woo DH (2013). Phosphorylation of EZH2 activates STAT3 signaling via STAT3 methylation and promotes tumorigenicity of glioblastoma stem-like cells.. Cancer Cell.

[123] Zhou J, Yi L, Ouyang Q (2014). Neurotensin signaling regulates stem-like traits of glioblastoma stem cells through activation of IL-8/CXCR1/STAT3 pathway.. Cell Signal.

[124] Swiatek-Machado K, Kaminska B (2020). STAT signaling in glioma cells.. Adv Exp Med Biol.

[125] Lo HW, Cao X, Zhu H, Ali-Osman F (2008). Constitutively activated STAT3 frequently coexpresses with epidermal growth factor receptor in high-grade gliomas and targeting STAT3 sensitizes them to Iressa and alkylators.. Clin Cancer Res.

[126] Krasilnikov M, Ivanov VN, Dong J, Ronai Z (2003). ERK and PI3K negatively regulate STAT-transcriptional activities in human melanoma cells: implications towards sensitization to apoptosis.. Oncogene.

[127] Lu Y, Zhou J, Xu C (2008). JAK/STAT and PI3K/AKT pathways form a mutual transactivation loop and afford resistance to oxidative stress-induced apoptosis in cardiomyocytes.. Cell Physiol Biochem.

[128] Sharfe N, Dadi HK, Roifman CM (1995). JAK3 protein tyrosine kinase mediates interleukin-7-induced activation of phosphatidylinositol-3’ kinase.. Blood.

[129] Kohsaka S, Wang L, Yachi K (2012). STAT3 inhibition overcomes temozolomide resistance in glioblastoma by downregulating MGMT expression.. Mol Cancer Ther.

[130] Dai B, Meng J, Peyton M (2011). STAT3 mediates resistance to MEK inhibitor through microRNA miR-17.. Cancer Res.

[131] Xu H, Lai W, Zhang Y (2014). Tumor-associated macrophage-derived IL-6 and IL-8 enhance invasive activity of LoVo cells induced by PRL-3 in a KCNN4 channel-dependent manner.. BMC Cancer.

[132] Wang Y, Chen L, Bao Z (2011). Inhibition of STAT3 reverses alkylator resistance through modulation of the AKT and beta-catenin signaling pathways.. Oncol Rep.

[133] Lee HH, Lin CH, Lin HY (2019). Histone 2A family member j drives mesenchymal transition and temozolomide resistance in glioblastoma multiforme.. Cancers (Basel).

[134] Caja L, Tzavlaki K, Dadras MS (2018). Snail regulates BMP and TGFbeta pathways to control the differentiation status of glioma-initiating cells.. Oncogene.

[135] Liang H, Chen G, Li J, Yang F (2019). Snail expression contributes to temozolomide resistance in glioblastoma.. Am J Transl Res.

[136] Cui P, Wei F, Hou J (2020). STAT3 inhibition induced temozolomide-resistant glioblastoma apoptosis via triggering mitochondrial STAT3 translocation and respiratory chain dysfunction.. Cell Signal.

[137] Nazio F, Bordi M, Cianfanelli V, Locatelli F, Cecconi F (2019). Autophagy and cancer stem cells: molecular mechanisms and therapeutic applications.. Cell Death Differ.

[138] Jiapaer S, Furuta T, Tanaka S, Kitabayashi T, Nakada M (2018). Potential strategies overcoming the temozolomide resistance for glioblastoma.. Neurol Med Chir (Tokyo).

[139] Klionsky DJ, Schulman BA (2014). Dynamic regulation of macroautophagy by distinctive ubiquitin-like proteins.. Nat Struct Mol Biol.

[140] He Y, Su J, Lan B, Gao Y, Zhao J (2019). Targeting off-target effects: endoplasmic reticulum stress and autophagy as effective strategies to enhance temozolomide treatment.. Onco Targets Ther.

[141] Paglin S, Hollister T, Delohery T (2001). A novel response of cancer cells to radiation involves autophagy and formation of acidic vesicles.. Cancer Res.

[142] Sui X, Chen R, Wang Z (2013). Autophagy and chemotherapy resistance: a promising therapeutic target for cancer treatment.. Cell Death Dis.

[143] Kanzawa T, Germano IM, Komata T (2004). Role of autophagy in temozolomide-induced cytotoxicity for malignant glioma cells.. Cell Death Differ.

[144] Carmo A, Carvalheiro H, Crespo I, Nunes I, Lopes MC (2011). Effect of temozolomide on the U-118 glioma cell line.. Oncol Lett.

[145] Hombach-Klonisch S, Mehrpour M, Shojaei S (2018). Glioblastoma and chemoresistance to alkylating agents: involvement of apoptosis, autophagy, and unfolded protein response.. Pharmacol Ther.

[146] Lin CJ, Lee CC, Shih YL (2012). Resveratrol enhances the therapeutic effect of temozolomide against malignant glioma in vitro and in vivo by inhibiting autophagy.. Free Radic Biol Med.

[147] Chen CM, Syu JP, Way TD (2015). BC3EE2,9B, a synthetic carbazole derivative, upregulates autophagy and synergistically sensitizes human GBM8901 glioblastoma cells to temozolomide.. Int J Mol Med.

[148] Filippi-Chiela EC, Bueno e Silva MM, Thome MP, Lenz G (2015). Single-cell analysis challenges the connection between autophagy and senescence induced by DNA damage.. Autophagy.

[149] Zou Y, Wang Q, Wang W (2015). MutL homolog 1 contributes to temozolomide-induced autophagy via ataxia-telangiectasia mutated in glioma.. Mol Med Rep.

[150] Sano R, Reed JC (2013). ER stress-induced cell death mechanisms.. Biochim Biophys Acta.

[151] Golden EB, Cho HY, Jahanian A (2014). Chloroquine enhances temozolomide cytotoxicity in malignant gliomas by blocking autophagy.. Neurosurg Focus.

[152] Zanotto-Filho A, Braganhol E, Klafke K (2015). Autophagy inhibition improves the efficacy of curcumin/temozolomide combination therapy in glioblastomas.. Cancer Lett.

[153] Wen ZP, Zeng WJ, Chen YH (2019). Knockdown ATG4C inhibits gliomas progression and promotes temozolomide chemosensitivity by suppressing autophagic flux.. J Exp Clin Cancer Res.

[154] Amaravadi RK, Thompson CB (2007). The roles of therapy-induced autophagy and necrosis in cancer treatment.. Clin Cancer Res.

[155] Deegan S, Saveljeva S, Logue SE (2014). Deficiency in the mitochondrial apoptotic pathway reveals the toxic potential of autophagy under ER stress conditions.. Autophagy.

[156] Zhang YB, Zhao W, Zeng RX (2013). Autophagic degradation of caspase-8 protects U87MG cells against H2O2-induced oxidative stress.. Asian Pac J Cancer Prev.

[157] Ye F, Zhang Y, Liu Y (2013). Protective properties of radio-chemoresistant glioblastoma stem cell clones are associated with metabolic adaptation to reduced glucose dependence.. PLoS One.

[158] Knizhnik AV, Roos WP, Nikolova T (2013). Survival and death strategies in glioma cells: autophagy, senescence and apoptosis triggered by a single type of temozolomide-induced DNA damage.. PLoS One.

[159] Johannessen TC, Hasan-Olive MM, Zhu H (2019). Thioridazine inhibits autophagy and sensitizes glioblastoma cells to temozolomide.. Int J Cancer.

[160] Josset E, Burckel H, Noel G, Bischoff P (2013). The mTOR inhibitor RAD001 potentiates autophagic cell death induced by temozolomide in a glioblastoma cell line.. Anticancer Res.

[161] Liu T, Li A, Xu Y, Xin Y (2019). Momelotinib sensitizes glioblastoma cells to temozolomide by enhancement of autophagy via JAK2/STAT3 inhibition.. Oncol Rep.

[162] Li C, Liu Y, Liu H (2015). Impact of autophagy inhibition at different stages on cytotoxic effect of autophagy inducer in glioblastoma cells.. Cell Physiol Biochem.

[163] Kim EL, Wustenberg R, Rubsam A (2010). Chloroquine activates the p53 pathway and induces apoptosis in human glioma cells.. Neuro Oncol.

[164] Rosenfeld MR, Ye X, Supko JG (2014). A phase I/II trial of hydroxychloroquine in conjunction with radiation therapy and concurrent and adjuvant temozolomide in patients with newly diagnosed glioblastoma multiforme.. Autophagy.

[165] Shao CJ, Wu MW, Chen FR (2012). Histone deacetylase inhibitor, 2-propylpentanoic acid, increases the chemosensitivity and radiosensitivity of human glioma cell lines in vitro.. Chin Med J (Engl).

[166] Lohitesh K, Saini H, Srivastava A (2018). Autophagy inhibition potentiates SAHAmediated apoptosis in glioblastoma cells by accumulation of damaged mitochondria.. Oncol Rep.

[167] Russell RC, Tian Y, Yuan H (2013). ULK1 induces autophagy by phosphorylating Beclin-1 and activating VPS34 lipid kinase.. Nat Cell Biol.

[168] Stjepanovic G, Baskaran S, Lin MG, Hurley JH (2017). Vps34 kinase domain dynamics regulate the autophagic PI 3-kinase complex.. Mol Cell.

[169] Chu CW, Ko HJ, Chou CH (2019). Thioridazine enhances P62-mediated autophagy and apoptosis through wnt/beta-catenin signaling pathway in glioma cells.. Int J Mol Sci.

[170] Yang MC, Loh JK, Li YY (2015). Bcl2L12 with a BH3-like domain in regulating apoptosis and TMZ-induced autophagy: a prospective combination of ABT-737 and TMZ for treating glioma.. Int J Oncol.

[171] Scarfo L, Ghia P (2013). Reprogramming cell death: BCL2 family inhibition in hematological malignancies.. Immunol Lett.

[172] Yan Y, Xu Z, Dai S (2016). Targeting autophagy to sensitive glioma to temozolomide treatment.. J Exp Clin Cancer Res.

[173] Torres S, Lorente M, Rodriguez-Fornes F (2011). A combined preclinical therapy of cannabinoids and temozolomide against glioma.. Mol Cancer Ther.

[174] Ulasov IV, Sonabend AM, Nandi S (2009). Combination of adenoviral virotherapy and temozolomide chemotherapy eradicates malignant glioma through autophagic and apoptotic cell death in vivo.. Br J Cancer.

[175] Lin CJ, Lee CC, Shih YL (2012). Inhibition of mitochondria- and endoplasmic reticulum stress-mediated autophagy augments temozolomide-induced apoptosis in glioma cells.. PLoS One.

[176] Seto E, Yoshida M (2014). Erasers of histone acetylation: the histone deacetylase enzymes.. Cold Spring Harb Perspect Biol.

[177] Xu WS, Parmigiani RB, Marks PA (2007). Histone deacetylase inhibitors: molecular mechanisms of action.. Oncogene.

[178] Gregoretti IV, Lee YM, Goodson HV (2004). Molecular evolution of the histone deacetylase family: functional implications of phylogenetic analysis.. J Mol Biol.

[179] Ropero S, Esteller M (2007). The role of histone deacetylases (HDACs) in human cancer.. Mol Oncol.

[180] Zhao LM, Zhang JH (2019). Histone deacetylase inhibitors in tumor immunotherapy.. Curr Med Chem.

[181] Sun Y, Sun Y, Yue S, Wang Y, Lu F (2018). Histone deacetylase inhibitors in cancer therapy.. Curr Top Med Chem.

[182] Bolden JE, Peart MJ, Johnstone RW (2006). Anticancer activities of histone deacetylase inhibitors.. Nat Rev Drug Discov.

[183] Carew JS, Giles FJ, Nawrocki ST (2008). Histone deacetylase inhibitors: mechanisms of cell death and promise in combination cancer therapy.. Cancer Lett.

[184] Bezecny P (2014). Histone deacetylase inhibitors in glioblastoma: pre-clinical and clinical experience.. Med Oncol.

[185] Yelton CJ, Ray SK (2018). Histone deacetylase enzymes and selective histone deacetylase inhibitors for antitumor effects and enhancement of antitumor immunity in glioblastoma.. Neuroimmunol Neuroinflamm.

[186] Lee DH, Ryu HW, Won HR, Kwon SH (2017). Advances in epigenetic glioblastoma therapy.. Oncotarget.

[187] Staberg M, Michaelsen SR, Rasmussen RD (2017). Inhibition of histone deacetylases sensitizes glioblastoma cells to lomustine.. Cell Oncol (Dordr).

[188] Li S, Chen X, Mao L (2018). Histone deacetylase 1 promotes glioblastoma cell proliferation and invasion via activation of PI3K/AKT and MEK/ERK signaling pathways.. Brain Res.

[189] Liu JR, Yu CW, Hung PY, Hsin LW, Chern JW (2019). High-selective HDAC6 inhibitor promotes HDAC6 degradation following autophagy modulation and enhanced antitumor immunity in glioblastoma.. Biochem Pharmacol.

[190] Santos-Barriopedro I, Li Y, Bahl S, Seto E (2019). HDAC8 affects MGMT levels in glioblastoma cell lines via interaction with the proteasome receptor ADRM1.. Genes Cancer.

[191] Kim GW, Lee DH, Yeon SK (2019). Temozolomide-resistant glioblastoma depends on HDAC6 activity through regulation of DNA mismatch repair.. Anticancer Res.

[192] Li ZY, Li QZ, Chen L (2016). Histone deacetylase inhibitor RGFP109 overcomes temozolomide resistance by blocking NF-kappaB-dependent transcription in glioblastoma cell lines.. Neurochem Res.

[193] Luo JW, Wang X, Yang Y, Mao Q (2015). Role of micro-RNA (miRNA) in pathogenesis of glioblastoma.. Eur Rev Med Pharmacol Sci.

[194] Novakova J, Slaby O, Vyzula R, Michalek J (2009). MicroRNA involvement in glioblastoma pathogenesis.. Biochem Biophys Res Commun.

[195] Bartel DP (2004). MicroRNAs: genomics, biogenesis, mechanism, and function.. Cell.

[196] Kirstein A, Schmid TE, Combs SE (2020). The role of miRNA for the treatment of MGMT unmethylated glioblastoma multiforme.. Cancers (Basel).

[197] Mizoguchi M, Guan Y, Yoshimoto K (2013). Clinical implications of microRNAs in human glioblastoma.. Front Oncol.

[198] Sarkar S, Ahmad A, Mittal S (2014). The therapeutic role of microRNAs in human gliomas. MicroRNA Targeted Cancer Therapy..

[199] Mathupala SP, Mittal S, Guthikonda M, Sloan AE (2007). MicroRNA and brain tumors: a cause and a cure?. DNA Cell Biol.

[200] Mathupala S, Mittal S, Guthikonda M, Sloan A (2011). RNAi-based approaches to the treatment of brain tumors. MicroRNAs in cancer translational research..

[201] Shi L, Chen J, Yang J (2010). MiR-21 protected human glioblastoma U87MG cells from chemotherapeutic drug temozolomide induced apoptosis by decreasing Bax/Bcl-2 ratio and caspase-3 activity.. Brain Res.

[202] Zhang S, Wan Y, Pan T (2012). MicroRNA-21 inhibitor sensitizes human glioblastoma U251 stem cells to chemotherapeutic drug temozolomide.. J Mol Neurosci.

[203] Zhou RJ, Xu XY, Liu BX (2015). Growth-inhibitory and chemosensitizing effects of microRNA-31 in human glioblastoma multiforme cells.. Int J Mol Med.

[204] Chen J, Fu X, Wan Y (2014). miR-125b inhibitor enhance the chemosensitivity of glioblastoma stem cells to temozolomide by targeting Bak1.. Tumour Biol.

[205] Haemmig S, Baumgartner U, Gluck A (2014). miR-125b controls apoptosis and temozolomide resistance by targeting TNFAIP3 and NKIRAS2 in glioblastomas.. Cell Death Dis.

[206] Shi L, Zhang S, Feng K (2012). MicroRNA-125b-2 confers human glioblastoma stem cells resistance to temozolomide through the mitochondrial pathway of apoptosis.. Int J Oncol.

[207] Shi L, Wan Y, Sun G (2014). miR-125b inhibitor may enhance the invasion-prevention activity of temozolomide in glioblastoma stem cells by targeting PIAS3.. BioDrugs.

[208] Chen H, Li X, Li W, Zheng H (2015). miR-130a can predict response to temozolomide in patients with glioblastoma multiforme, independently of O6-methylguanine-DNA methyltransferase.. J Transl Med.

[209] Zhang HD, Jiang LH, Sun DW, Li J, Ji ZL (2017). The role of miR-130a in cancer.. Breast Cancer.

[210] Wong ST, Zhang XQ, Zhuang JT (2012). MicroRNA-21 inhibition enhances in vitro chemosensitivity of temozolomide-resistant glioblastoma cells.. Anticancer Res.

[211] Chen L, Zhang J, Han L (2012). Downregulation of miR-221/222 sensitizes glioma cells to temozolomide by regulating apoptosis independently of p53 status.. Oncol Rep.

[212] Brognara E, Fabbri E, Montagner G (2016). High levels of apoptosis are induced in human glioma cell lines by co-administration of peptide nucleic acids targeting miR-221 and miR-222.. Int J Oncol.

[213] Quintavalle C, Mangani D, Roscigno G (2013). MiR-221/222 target the DNA methyltransferase MGMT in glioma cells.. PLoS One.

[214] She X, Yu Z, Cui Y (2014). miR-128 and miR-149 enhance the chemosensitivity of temozolomide by Rap1B-mediated cytoskeletal remodeling in glioblastoma.. Oncol Rep.

[215] Lee D, Sun S, Zhang XQ (2015). MicroRNA-210 and Endoplasmic Reticulum Chaperones in the Regulation of Chemoresistance in Glioblastoma.. J Cancer.

[216] Chen G, Chen Z, Zhao H (2020). MicroRNA-155-3p promotes glioma progression and temozolomide resistance by targeting Six1.. J Cell Mol Med.

[217] Lee YY, Yarmishyn AA, Wang ML (2018). MicroRNA-142-3p is involved in regulation of MGMT expression in glioblastoma cells.. Cancer Manag Res.

[218] Chiou GY, Chien CS, Wang ML (2013). Epigenetic regulation of the miR142-3p/interleukin-6 circuit in glioblastoma.. Mol Cell.

[219] Zhang W, Zhang J, Hoadley K (2012). miR-181d: a predictive glioblastoma biomarker that downregulates MGMT expression.. Neuro Oncol.

[220] Nadaradjane A, Briand J, Bougras-Cartron G (2018). miR-370-3p is a therapeutic tool in anti-glioblastoma therapy but is not an intratumoral or cell-free circulating biomarker.. Mol Ther Nucleic Acids.

[221] Gao YT, Chen XB, Liu HL (2016). Up-regulation of miR-370-3p restores glioblastoma multiforme sensitivity to temozolomide by influencing MGMT expression.. Sci Rep.

[222] Khalil S, Fabbri E, Santangelo A (2016). miRNA array screening reveals cooperative MGMT-regulation between miR-181d-5p and miR-409-3p in glioblastoma.. Oncotarget.

[223] Kushwaha D, Ramakrishnan V, Ng K (2014). A genome-wide miRNA screen revealed miR-603 as a MGMT-regulating miRNA in glioblastomas.. Oncotarget.

[224] Kreth S, Limbeck E, Hinske LC (2013). In human glioblastomas transcript elongation by alternative polyadenylation and miRNA targeting is a potent mechanism of MGMT silencing.. Acta Neuropathol.

[225] Ren Y, Zhou X, Mei M (2010). MicroRNA-21 inhibitor sensitizes human glioblastoma cells U251 (PTEN-mutant) and LN229 (PTEN-wild type) to taxol.. BMC Cancer.

[226] Zhang S, Han L, Wei J (2015). Combination treatment with doxorubicin and microRNA-21 inhibitor synergistically augments anticancer activity through upregulation of tumor suppressing genes.. Int J Oncol.

[227] Barker CA, Chang M, Chou JF (2012). Radiotherapy and concomitant temozolomide may improve survival of elderly patients with glioblastoma.. J Neurooncol.

[228] Costa PM, Cardoso AL, Nobrega C (2013). MicroRNA-21 silencing enhances the cytotoxic effect of the antiangiogenic drug sunitinib in glioblastoma.. Hum Mol Genet.

[229] Li Y, Li W, Yang Y (2009). MicroRNA-21 targets LRRFIP1 and contributes to VM-26 resistance in glioblastoma multiforme.. Brain Res.

[230] Wang Z, Li Z, Fu Y, Han L, Tian Y (2019). MiRNA-130a-3p inhibits cell proliferation, migration, and TMZ resistance in glioblastoma by targeting Sp1.. Am J Transl Res.

[231] Tan Z, Jia J, Jiang Y (2018). MiR-150-3p targets SP1 and suppresses the growth of glioma cells.. Biosci Rep.

[232] Chuang JY, Lo WL, Ko CY (2017). Upregulation of CYP17A1 by Sp1-mediated DNA demethylation confers temozolomide resistance through DHEA-mediated protection in glioma.. Oncogenesis.

[233] Yang YN, Zhang XH, Wang YM, Zhang X, Gu Z (2018). miR-204 reverses temozolomide resistance and inhibits cancer initiating cells phenotypes by degrading FAP-alpha in glioblastoma.. Oncol Lett.

[234] Shea A, Harish V, Afzal Z (2016). MicroRNAs in glioblastoma multiforme pathogenesis and therapeutics.. Cancer Med.

[235] Comincini S, Allavena G, Palumbo S (2013). microRNA-17 regulates the expression of ATG7 and modulates the autophagy process, improving the sensitivity to temozolomide and low-dose ionizing radiation treatments in human glioblastoma cells.. Cancer Biol Ther.

[236] Namee NM, O’Driscoll L (2018). Extracellular vesicles and anti-cancer drug resistance.. Biochim Biophys Acta Rev Cancer.

[237] Saadatpour L, Fadaee E, Fadaei S (2016). Glioblastoma: exosome and microRNA as novel diagnosis biomarkers.. Cancer Gene Ther.

[238] D’Asti E, Chennakrishnaiah S, Lee TH, Rak J (2016). Extracellular vesicles in brain tumor progression.. Cell Mol Neurobiol.

[239] Gourlay J, Morokoff AP, Luwor RB (2017). The emergent role of exosomes in glioma.. J Clin Neurosci.

[240] Zeng A, Wei Z, Yan W (2018). Exosomal transfer of miR-151a enhances chemosensitivity to temozolomide in drug-resistant glioblastoma.. Cancer Lett.

[241] Zhang Z, Yin J, Lu C (2019). Exosomal transfer of long non-coding RNA SBF2-AS1 enhances chemoresistance to temozolomide in glioblastoma.. J Exp Clin Cancer Res.

[242] Basu B, Ghosh MK (2019). Extracellular vesicles in glioma: from diagnosis to therapy.. Bioessays.

[243] Zeng AL, Yan W, Liu YW (2017). Tumour exosomes from cells harbouring PTPRZ1-MET fusion contribute to a malignant phenotype and temozolomide chemoresistance in glioblastoma.. Oncogene.

[244] Kore RA, Edmondson JL, Jenkins SV (2018). Hypoxia-derived exosomes induce putative altered pathways in biosynthesis and ion regulatory channels in glioblastoma cells.. Biochem Biophys Rep.

[245] Akers JC, Ramakrishnan V, Kim R (2013). MiR-21 in the extracellular vesicles (EVs) of cerebrospinal fluid (CSF): a platform for glioblastoma biomarker development.. PLoS One.

[246] Shao H, Chung J, Lee K (2015). Chip-based analysis of exosomal mRNA mediating drug resistance in glioblastoma.. Nat Commun.

[247] Yekula A, Taylor A, Beecroft A (2020). The role of extracellular vesicles in acquisition of resistance to therapy in glioblastomas.. Cancer Drug Resist.

